# Supplementary material for: Identification of Freezing-Responsive microRNAs and Their Targets in Chinese Jujube by Small RNA and Degradome Sequencing
Source: Int J Mol Sci. 2026 Sep 4;27(17):7912. doi: 10.3390/ijms27177912 (PMC13566890; doi:10.3390/ijms27177912)
Supplement: Supplementary file 1 [file ijms-27-07912-s001.zip › Supplementary Material S2/novel_miR_21.pdf]

Provisional ID	:	novel_miR_21
Score total	:	0.6
Score for star read(s)	:	-1.3
Score for read counts	:	0
Score for mfe	:	0.3
Score for randfold	:	1.6
Score for cons. seed	:	
Total read count	:	140253
Mature read count	:	140253
Loop read count	:	0
Star read count	:	0

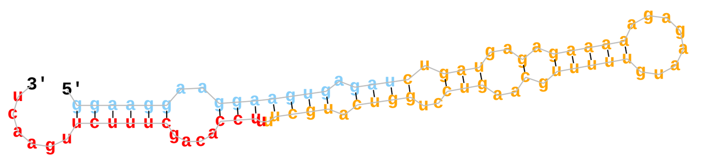

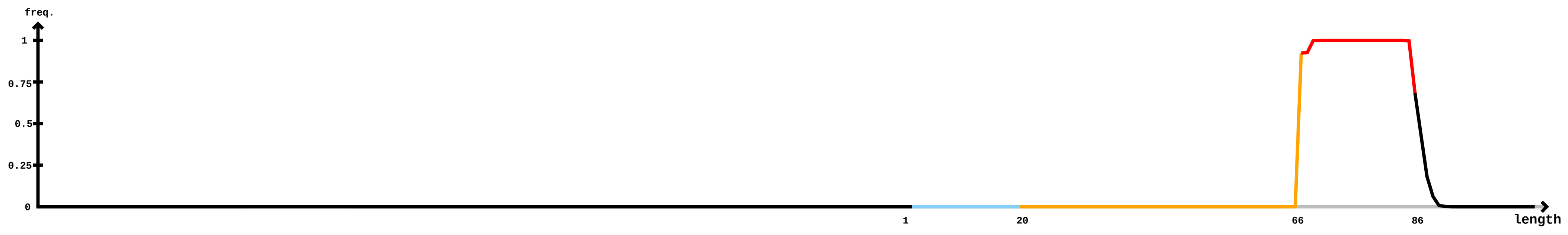

Star **Mature**

```
5'- ucucuuucucucucucucucucuaaaaaaacaaaaggaaagaaaaaaaaaaaaaaaaacuuacuacuacuacuaguaguauaucuucuaauuugcuggaaaggcacaccaaagagaaagaaauuaaaccaaggaaggaaggaaggaaggaaggaagugagaucugaugagagaaaaagagaauguuuuugcaaguccuggucaugcuuuuccacagcuuucuugaacuuucuuucuuucuguuuagucu -3'
    ..((((((((.....(((.(((...........))).)))................((((((((....))))))))...........((((.....)))).....))))))))).((((((..((((((((((((((..(((((((..((((((.(((((.((.((....((((....)))).....)))).)))).)..))).)))..)))))))...))))))))))))))))))).. 
```

Read	exp reads	mm	sample
ucucuuucuGucucucuc......	5	1	LT-2
...cuuuGucucucucucucucu......	1	1	LT-3
.......cucucucucuUucucuaa......	1	1	LT-1
......Aaaaaggaaagaaaaaaaaaa......	5	1	LCK-3
......aaaaggaaagaaaaaaaaaaaaa......	1	0	JT-1
......aaaaggaaagaaaaaaaaaaaaaaa......	1	0	JT-1
......aUggaaagaaaaaaaaaaa......	1	1	LT-1
......aggaaGgaaaaaaaaaaaaaaaaaa......	1	1	LT-3
......aNaaaaaaaaaaaaaaaaa......	1	1	JCK-3
......uuuuccacagcuuucuugaac......	3	0	JT-3
......uuuuccacagcuuucuugaac......	6	0	JCK-2
......uuuuccacagcuuucuugaac......	3	0	LCK-3
......uuuuccacagcuuucuugaac......	7	0	JCK-3
......uuuuccacagcuuucuugaac......	4	0	JCK-1
......uuuuccacagcuuucuugaac......	3	0	LT-3
......uuuuccacagcuuucuugaac......	2	0	JT-2
......uuuuccacagcuuucuugaac......	3	0	LT-1
......uuuuccacagcuuucuugaacu......	1	0	JCK-3
......uuuuccacagcuuucuugaacu......	4	0	JCK-2
......uNuuccacagcuuucuugaacu......	1	1	JCK-3
......uuuccacagcuuucuuga......	2	0	JCK-1
......uuuccacagcuuucuugaacu......	1	0	JCK-3
......uuuccacagcuuucuugaacu......	2	0	LCK-3
......Auuccacagcuuucuugaacu......	1	1	JCK-3
......Guuccacagcuuucuugaacu......	2	1	LT-3
......uuuccacagcuuucuugaacu......	1	0	JCK-2
......Auuccacagcuuucuugaacu......	2	1	JCK-2
......uuuccacagcuuucuugaacu......	1	0	LT-1
......Cuuccacagcuuucuugaacuu......	2	1	JCK-1
......Cuuccacagcuuucuugaacuu......	3	1	LT-1
......Cuuccacagcuuucuugaacuu......	1	1	LT-2
......Cuuccacagcuuucuugaacuu......	3	1	LT-3
......Cuuccacagcuuucuugaacuu......	3	1	JCK-2
......Cuuccacagcuuucuugaacuuu......	1	1	LT-3

Star Mature

```
ucucuuucucucucucucucucucuaaaaaaacaaaaggaaagaaaaaaaaaaaaaaaaacuuacuacuacuacuaguaguauaucuucuaauuugcuggaaaggcacaccaaagagaaagaaauuaaaccaaggaaggaaggaaggaaggaaggaagugagaucugaugagagaaaaagagaauguuuuugcaaguccuggucaugcuuuuccacagcuuucuugaacuuucuuucuuucuguuuagucu
.........................Cuuccacagcuuucuugaacuuu...................   2     1   JCK-1
.........................Cuuccacagcuuucuugaacuuu...................   1     1   LT-1
.........................Cuuccacagcuuucuugaacuuu...................   3     1   LCK-3
..........................uuccacagcuuucuugaa.......................   15    0   JCK-2
..........................uuccacagcuuucuugaa.......................   31    0   LT-1
..........................uuccacagcuuucuugaU.......................   3     1   LT-2
..........................uuccacagcuuucuugaa.......................   43    0   LCK-3
..........................uuccacagcuuucuugaa.......................   16    0   JCK-1
..........................uuccacagcuuucuugaC.......................   4     1   LT-2
..........................uuccacagcuuucuugUa.......................   19    1   LCK-3
..........................uuccacagcuuucuugaa.......................   9     0   JCK-3
..........................uuccacagcuuucuugaU.......................   4     1   LCK-3
..........................uuccacagcuuucuugaa.......................   46    0   LCK-1
..........................uuccacagcuuucuugaU.......................   11    1   LT-1
..........................uuccacagcuuucuugaC.......................   1     1   LT-3
..........................uuccacagcuuucuugGa.......................   1     1   LCK-2
..........................uuccacagcuuucuuUaa.......................   1     1   LT-1
..........................uuccacagcuuucuugaa.......................   52    0   LT-3
..........................uuccacagcuuucuugaU.......................   1     1   JCK-2
..........................uuccacagcuuucuugCa.......................   1     1   LT-2
..........................uuccacagcuuucuugUa.......................   1     1   LT-1
..........................uuccacagcuuucuugaa.......................   43    0   LT-2
..........................uuccacagcuuucuugaa.......................   53    0   LCK-2
..........................uuccacagcuuucuugaU.......................   1     1   JCK-1
..........................uuccacagcuuucuugaa.......................   5     0   JT-2
..........................uuccacagcuuucuugUa.......................   15    1   LT-2
..........................uuccacagcuuucuGgaa.......................   1     1   LCK-1
..........................uuccacagcuuucuugaa.......................   15    0   JT-3
..........................uuccacagcuuucuugaU.......................   14    1   LT-3
..........................uuccaNagcuuucuugaa.......................   1     1   LCK-1
..........................uuccacagcuuucuugUa.......................   4     1   LT-3
..........................uuccacagcuuucuugaC.......................   1     1   JCK-3
..........................uuccacagcuuucuugUa.......................   2     1   JCK-3
..........................uuccacagcuuucuugUa.......................   5     1   JT-1
..........................uNccacagcuuucuugaac......................   2     1   JCK-3
..........................uuccacagcuCucuugaac......................   2     1   JCK-2
..........................uuccacagcuuucuugaGc......................   1     1   JCK-1
..........................uuccCcagcuuucuugaac......................   4     1   JCK-2
..........................uuccacagcuuucuugaCc......................   2     1   LCK-2
..........................uuccacCgcuuucuugaac......................   16    1   LT-3
..........................uuccCcagcuuucuugaac......................   2     1   LCK-1
..........................uCccacagcuuucuugaac......................   1     1   LCK-1
..........................uuccacagcuuucuugaGc......................   1     1   LCK-3
..........................uuccGcagcuuucuugaac......................   1     1   LT-1
..........................uuccacagcuuucuugaCc......................   8     1   LCK-3
..........................uuccacagcuuucuugaaU......................   24    1   LCK-2
..........................uuccacagcuuucuNgaac......................   4     1   LCK-1
..........................uuccacagcuGucuugaac......................   1     1   LT-1
..........................uuccacagcuuucuugaCc......................   10    1   LT-3
..........................uuccaUagcuuucuugaac......................   2     1   LT-3
..........................uuccCcagcuuucuugaac......................   1     1   JCK-1
..........................uuccacagcuuucuugCac......................   4     1   LT-1
..........................uuccCcagcuuucuugaac......................   1     1   JT-3
..........................uuccacagcuuucuugaac......................   1739  0   LCK-2
..........................uuccacaAcuuucuugaac......................   1     1   JCK-3
..........................uuccacagcuuucuugaaU......................   64    1   LT-2
..........................uuccacagcuuucuugaaU......................   26    1   LCK-1
..........................uuccacagcuuuUuugaac......................   8     1   JCK-2
..........................uuccacaAcuuucuugaac......................   1     1   JCK-2
..........................uuccacagcuuucuugCac......................   1     1   LCK-1
..........................uuccacagUuuucuugaac......................   4     1   LCK-3
..........................uuccaUagcuuucuugaac......................   1     1   LT-2
..........................uuccacagcuuucuugaaU......................   98    1   LT-3
..........................uuccacagcuuucuugaGc......................   1     1   LT-3
..........................uuccacagcuuCcuugaac......................   2     1   JCK-3
..........................uuccacagcuuucuugaac......................   2667  0   JCK-3
..........................uuccacagcuuucuugaCc......................   1     1   LCK-1
..........................uuccacagcuuucuugCac......................   3     1   JT-1
..........................uuccacaUcuuucuugaac......................   2     1   JCK-2
..........................uuccacagcuuucCugaac......................   2     1   LCK-3
```

```
                                                                                                                                      Star                                          Mature
ucucuuucucucucucucucucuaaaaaaacaaaaggaaagaaaaaaaaaaaaaaaaaaacuuacuacuacuacuaguaguauaucuucuaauuugcuggaaaggcacaccaaagagaaagaaauuaaaccaaggaaggaaggaaggaaggaaggaagugagaucugaugagagaaaaagagaauguuuuugcaaguccuggucaugcuuuuccacagcuuucuugaacuuucuuucuuucuguuuagucu
...........uuccacagcuuucuugaUc.....................	3	1	JT-1
...........uuccacagcuuucuugaCc.....................	5	1	LT-1
...........uucAacagcuuucuugaac.....................	1	1	LT-2
...........uuccacagcuuucuugaac.....................	1676	0	LCK-1
...........uuccacagcuuucuuAaac.....................	1	1	JCK-1
...........uuccacagcuuucuugaac.....................	2180	0	JCK-1
...........uuccacagcuuucNugaac.....................	6	1	LCK-1
...........uuUcacagcuuucuugaac.....................	1	1	LT-3
...........uuccacagcuuucuugCac.....................	5	1	JCK-2
...........uuccacaAcuuucuugaac.....................	5	1	LT-1
...........uuccaAagcuuucuugaac.....................	1	1	JCK-1
...........uuccacagcCuucuugaac.....................	1	1	LT-2
...........Cuccacagcuuucuugaac.....................	2	1	JT-1
...........Cuccacagcuuucuugaac.....................	1	1	JCK-3
...........uuccacagcuuNcuugaac.....................	2	1	JCK-1
...........uucUacagcuuucuugaac.....................	4	1	JCK-2
...........uNccacagcuuucuugaac.....................	5	1	JCK-2
...........uuccacaAcuuucuugaac.....................	2	1	LT-2
...........uuccacCgcuuucuugaac.....................	9	1	JCK-2
...........uuccaAagcuuucuugaac.....................	1	1	LCK-3
...........uuccacagcuuucuugaUc.....................	2	1	JCK-1
...........uuccacCgcuuucuugaac.....................	10	1	LT-2
...........uuccacagcuuuAuugaac.....................	1	1	LT-2
...........uuccacagcuuCcuugaac.....................	2	1	LT-1
...........uuccacagcuuucuugCac.....................	1	1	LCK-2
...........uuccacaAcuuucuugaac.....................	2	1	LCK-3
...........uNccacagcuuucuugaac.....................	3	1	JT-1
...........uuccacagcCuucuugaac.....................	1	1	JCK-2
...........Cuccacagcuuucuugaac.....................	3	1	LT-3
...........uuccacagcuuucuGgaac.....................	1	1	JCK-2
...........uuccacagcuuucuugGac.....................	1	1	JCK-3
...........uuccacagcuuucCugaac.....................	3	1	JCK-2
...........uuccaAagcuuucuugaac.....................	1	1	LT-2
...........uuccacagcuuuNuugaac.....................	1	1	LCK-1
...........uuccacagcuuucuugaCc.....................	7	1	JCK-2
...........uuccacagcuuucuugCac.....................	1	1	JCK-3
...........uuccacagcuuucuugaaU.....................	103	1	LCK-3
...........uuccaUagcuuucuugaac.....................	1	1	JCK-2
...........uuccCcagcuuucuugaac.....................	1	1	LCK-2
...........uuccacagcuGucuugaac.....................	3	1	JCK-1
...........uuccacagcuuucuugaCc.....................	1	1	JCK-3
...........uuccacagUuuucuugaac.....................	3	1	LT-2
...........uCccacagcuuucuugaac.....................	1	1	JCK-1
...........uuccacCgcuuucuugaac.....................	12	1	LT-1
...........uuccacagcCuucuugaac.....................	7	1	LT-1
...........uucUacagcuuucuugaac.....................	2	1	JCK-1
...........uuccacagcuuucuugaUc.....................	10	1	LCK-3
...........uuccacagUuuucuugaac.....................	1	1	LT-3
...........uuccacCgcuuucuugaac.....................	2	1	JT-1
...........uuUcacagcuuucuugaac.....................	2	1	JCK-2
...........uuccacagcuuuUuugaac.....................	1	1	LCK-2
...........uuccCcagcuuucuugaac.....................	33	1	LT-2
...........uuccacagcCuucuugaac.....................	1	1	JCK-1
...........uuccacaAcuuucuugaac.....................	1	1	LT-3
...........uuccacCgcuuucuugaac.....................	2	1	LCK-2
...........uuccacagcuuuAuugaac.....................	2	1	LT-1
...........uuccacCgcuuucuugaac.....................	1	1	JT-2
...........uuccacagcuuucuugCac.....................	3	1	LCK-3
...........uuccacagcuuucuugaac.....................	1423	0	JT-1
...........uuccacagcuuucuugaac.....................	5694	0	LT-2
...........uuccacagcuuCcuugaac.....................	3	1	LCK-3
...........uuccacagcuuuUuugaac.....................	4	1	JCK-1
...........uuccacagcuuucuugaac.....................	8638	0	LT-3
...........uuccacagcuuucuuAaac.....................	4	1	LT-2
...........uuccacagcuuucuugaCc.....................	13	1	LT-2
...........uuccaAagcuuucuugaac.....................	1	1	JCK-2
...........uuccacagcuuucCugaac.....................	1	1	LT-2
...........uuccacagcuuCcuugaac.....................	3	1	JCK-2
...........uuccaAagcuuucuugaac.....................	3	1	LT-3
...........uCccacagcuuucuugaac.....................	1	1	JCK-2
```

Star Mature

ucucuuucucucucucucucucuaaaaaaacaaaaggaaagaaaaaaaaaaaaaaaaaacuuacuacuacuacuaguaguauaucuucuaauuugcuggaaaggcacaccaaagagaaagaaauuaaaccaaggaaggaaggaaggaaggaaggaagugagaucugaugagagaaaaagagaauguuuuugcaaguccuggucaugcuuuuccacagcuuucuugaacuuucuuucuuucuguuuagucu

...uuccacCgcuuucuugaac...	2	1	JCK-1
...uuccacagcuuucuugCac...	11	1	LT-3
...uuccacagcuuucuugaUc...	3	1	LT-2
...uuUcacagcuuucuugaac...	1	1	JCK-3
...Cuccacagcuuucuugaac...	3	1	LCK-3
...uuccacagcuuuAuugaac...	2	1	LCK-3
...uuccacagcuuuUuugaac...	2	1	JCK-3
...uuccacagcuuucuugaaU...	37	1	LT-1
...uuccacCgcuuucuugaac...	11	1	LCK-3
...uuUcacagcuuucuugaac...	1	1	LT-1
...uuccacagcCuucuugaac...	5	1	LT-3
...uuccacagAuuucuugaac...	1	1	LT-2
...uuccacCgcuuucuugaac...	2	1	JCK-3
...uuccacagcuuucuugaUc...	2	1	LT-3
...uNccacagcuuucuugaac...	1	1	LCK-2
...uuccacagcuuucuugaaU...	24	1	JCK-1
...Cuccacagcuuucuugaac...	1	1	LT-2
...uNccacagcuuucuugaac...	1	1	JCK-1
...uuccCcagcuuucuugaac...	12	1	LT-1
...uuccacagcuuucGugaac...	3	1	LCK-1
...uuNcacagcuuucuugaac...	1	1	LCK-1
...uuccacagcuuucuCgaac...	1	1	LT-1
...uuccacagUuuucuugaac...	5	1	LT-1
...uuccacagcuuucuugaac...	3571	0	JCK-2
...uuccacagcuuucuugCac...	2	1	JCK-1
...uuccacagcuCucuugaac...	1	1	LT-1
...uuccacagcuuuUuugaac...	6	1	LT-2
...uuccacagcuuucCugaac...	2	1	LT-1
...uucUacagcuuucuugaac...	1	1	JCK-3
...uucNacagcuuucuugaac...	1	1	JCK-1
...uuccacagcuuucuGgaac...	2	1	LCK-1
...uuccacagcuuucuugaaU...	53	1	JCK-2
...uuccacagcuuucuugaac...	8415	0	LCK-3
...uuccCcagcuuucuugaac...	2	1	JCK-3
...uuccacagcuuucuuUaac...	1	1	JT-1
...uuccacagcuuucuugCac...	5	1	LT-2
...uuccaNagcuuucuugaac...	3	1	LCK-1
...uuccacagcuuucuugaCc...	1	1	JT-1
...uuAcacagcuuucuugaac...	1	1	JCK-1
...uuAcacagcuuucuugaac...	1	1	LT-3
...uuccacagcuuucuugUac...	1	1	LT-3
...uuccacagcuuucuugaac...	542	0	JT-3
...uCccacagcuuucuugaac...	2	1	LT-3
...uuccCcagcuuucuugaac...	16	1	LT-3
...uuccacGgcuuucuugaac...	2	1	JCK-2
...uuccacagcuuCcuugaac...	3	1	JCK-1
...uuccCcagcuuucuugaac...	2	1	JT-1
...uuccacagcuuucuugUac...	3	1	LCK-3
...uuccacagcuuucuugaaU...	28	1	JCK-3
...uuccacagcuuuUuugaac...	3	1	LT-1
...uuccacagcuuucuugaac...	6061	0	LT-1
...uuGcacagcuuucuugaac...	3	1	LT-2
...uuccacagcuuucuugaac...	411	0	JT-2
...uuccacagcuuucCugaac...	1	1	JCK-3
...Cuccacagcuuucuugaac...	4	1	LT-1
...uuUcacagcuuucuugaac...	6	1	JCK-1
...uuccCcagcuuucuugaac...	13	1	LCK-3
...uuNcacagcuuucuugaac...	14	1	LT-2
...uuccacagcuuucuugCacu...	3	1	JCK-1
...uuccacagcuuucuugaNcu...	1	1	LCK-1
...uuccacagcuuuAuugaacu...	1	1	LT-2
...uuccGcagcuuucuugaacu...	1	1	LCK-3
...uNccacagcuuucuugaacu...	2	1	LCK-1
...uuccacagcuuucuugCacu...	6	1	LT-3
...uuccUcagcuuucuugaacu...	1	1	JCK-2
...uuccacagcuuucuGgaacu...	1	1	JT-1
...uuccacCgcuuucuugaacu...	3	1	JCK-1
...uuccacagUuuucuugaacu...	1	1	JT-1
...uuccacagcuuucuuAaacu...	1	1	JCK-1
...uuccaAagcuuucuugaacu...	3	1	LT-1

```
                                                                                                                                                                                      Star                                                                                   Mature
ucucuuucucucucucucucucucuaaaaaaaacaaaaggaaagaaaaaaaaaaaaaaaaaacuuacuacuacuacuaguaguauaucuucuaauuugcuggaaaggcacaccaaagagaaagaaauuaaaccaaggaaggaaggaaggaaggaaggaagugagaucugaugagagaaaaagagaauguuuuugcaaguccuggucaugcuuuuccacagcuuucuugaacuuucuuucuuucuguuuagucu
....................................................................................................................................................................................................................uuccacGgcuuucuugaacu.....................    2      1    JT-3
....................................................................................................................................................................................................................uuccacagcuuucuugaaUu.....................    45     1    JT-2
....................................................................................................................................................................................................................uuccUcagcuuucuugaacu.....................    1      1    JCK-1
....................................................................................................................................................................................................................uuccacagcCuucuugaacu.....................    1      1    JT-3
....................................................................................................................................................................................................................uuccacagcuuucuugGacu.....................    1      1    LT-3
....................................................................................................................................................................................................................uuccacagcuuuUuugaacu.....................    1      1    LT-3
....................................................................................................................................................................................................................uuccacagcuuucuugaaAu.....................    2      1    LT-3
....................................................................................................................................................................................................................uuccacagcuuucuugaacu.....................    2309   0    JCK-3
....................................................................................................................................................................................................................uuccacagcuuucuugaaNu.....................    1      1    LT-1
....................................................................................................................................................................................................................uuccacagcuuucuugaacA.....................    11     1    JT-3
....................................................................................................................................................................................................................uuccacCgcuuucuugaacu.....................    1      1    JT-1
....................................................................................................................................................................................................................uuccaAagcuuucuugaacu.....................    1      1    LCK-1
....................................................................................................................................................................................................................uuccCcagcuuucuugaacu.....................    8      1    LT-3
....................................................................................................................................................................................................................uuccacagcuuucuugaacu.....................    2055   0    JT-1
....................................................................................................................................................................................................................uuccacagcuuucuCgaacu.....................    1      1    LT-2
....................................................................................................................................................................................................................uuccacagcuuucCugaacu.....................    1      1    JT-1
....................................................................................................................................................................................................................uNccacagcuuucuugaacu.....................    1      1    JCK-2
....................................................................................................................................................................................................................uuccacagcuuucuCgaacu.....................    1      1    LT-3
....................................................................................................................................................................................................................uuccacagcCuucuugaacu.....................    1      1    JCK-3
....................................................................................................................................................................................................................uuccacagcuuucuugaCcu.....................    1      1    JCK-3
....................................................................................................................................................................................................................uuccacagcuuCcuugaacu.....................    1      1    LCK-3
....................................................................................................................................................................................................................uuccacagcuuucuugaacG.....................    45     1    LCK-3
....................................................................................................................................................................................................................uuccacagcuCucuugaacu.....................    1      1    LCK-3
....................................................................................................................................................................................................................uuccacagcuuucuugCacu.....................    10     1    LT-1
....................................................................................................................................................................................................................uuccacagcuuucuugaacu.....................    7087   0    LT-3
....................................................................................................................................................................................................................uuccacCgcuuucuugaacu.....................    4      1    LT-1
....................................................................................................................................................................................................................uuNcacagcuuucuugaacu.....................    2      1    LCK-3
....................................................................................................................................................................................................................uuccacagcuuCcuugaacu.....................    2      1    LT-3
....................................................................................................................................................................................................................uuUcacagcuuucuugaacu.....................    1      1    JCK-3
....................................................................................................................................................................................................................uuccacagcuuucuugaaUu.....................    50     1    JT-3
....................................................................................................................................................................................................................uuccCcagcuuucuugaacu.....................    1      1    JCK-2
....................................................................................................................................................................................................................uuccacagcuuucuugaacC.....................    3      1    JT-2
....................................................................................................................................................................................................................uuccacagcuuucuugaacN.....................    2      1    JCK-1
....................................................................................................................................................................................................................uuccacagcuuucuugaCcu.....................    1      1    JT-2
....................................................................................................................................................................................................................uuccacagcuuuUuugaacu.....................    2      1    LT-1
....................................................................................................................................................................................................................uuccacagcuuucuugaaAu.....................    2      1    LT-1
....................................................................................................................................................................................................................uuccacagcuuucuugaacA.....................    48     1    JCK-1
....................................................................................................................................................................................................................uuccacagcCuucuugaacu.....................    1      1    JCK-1
....................................................................................................................................................................................................................uuccaAagcuuucuugaacu.....................    2      1    JCK-1
....................................................................................................................................................................................................................uCccacagcuuucuugaacu.....................    2      1    JCK-1
....................................................................................................................................................................................................................uuccacagcuuucuugaCcu.....................    11     1    LCK-3
....................................................................................................................................................................................................................uuccacagUuuucuugaacu.....................    1      1    LCK-3
....................................................................................................................................................................................................................uuccaAagcuuucuugaacu.....................    1      1    JCK-3
....................................................................................................................................................................................................................uuccacagcuGucuugaacu.....................    1      1    JCK-1
....................................................................................................................................................................................................................uuccacagUuuucuugaacu.....................    1      1    LT-1
....................................................................................................................................................................................................................uuccacagcuuucuugaacu.....................    10755  0    LCK-3
....................................................................................................................................................................................................................uuccacagcuuucuugaaUu.....................    154    1    LT-2
....................................................................................................................................................................................................................uuccacagcuCucuugaacu.....................    3      1    LT-1
....................................................................................................................................................................................................................uuccacagcuuucCugaacu.....................    1      1    LT-1
....................................................................................................................................................................................................................uuccacCgcuuucuugaacu.....................    3      1    LCK-2
....................................................................................................................................................................................................................uuccacagcuuucuugaacC.....................    5      1    JCK-3
....................................................................................................................................................................................................................uuccacagcuuucuugaacC.....................    2      1    JCK-2
....................................................................................................................................................................................................................uuccacagcuuucuugaaAu.....................    1      1    JCK-3
....................................................................................................................................................................................................................uuccacagcuuucuugaacu.....................    2125   0    JCK-1
....................................................................................................................................................................................................................uuccacagcuuucuugaCcu.....................    25     1    LT-2
....................................................................................................................................................................................................................uuccacagcuuNcuugaacu.....................    3      1    JT-1
....................................................................................................................................................................................................................uuccacagcuuucuugaacu.....................    544    0    JT-2
....................................................................................................................................................................................................................uuccacagcuuucuugGacu.....................    1      1    LT-2
....................................................................................................................................................................................................................uuccacagcuuucuugaacu.....................    731    0    JT-3
....................................................................................................................................................................................................................uuccacagcuuucuugaaUu.....................    192    1    LCK-3
....................................................................................................................................................................................................................uuccacagcuuucuugaUcu.....................    2      1    LT-2
....................................................................................................................................................................................................................uuccacagcuuucuugaacC.....................    8      1    JT-1
....................................................................................................................................................................................................................uuccacagcuuucuGgaacu.....................    4      1    LCK-1
....................................................................................................................................................................................................................uuccacagcuuucuuAaacu.....................    3      1    LCK-3
....................................................................................................................................................................................................................uCccacagcuuucuugaacu.....................    2      1    JCK-2
....................................................................................................................................................................................................................uuccacagcuuucuugaacu.....................    3389   0    JCK-2
....................................................................................................................................................................................................................uuccacagcuGucuugaacu.....................    1      1    LT-2
....................................................................................................................................................................................................................uuccacaAcuuucuugaacu.....................    1      1    LCK-2
....................................................................................................................................................................................................................uCccacagcuuucuugaacu.....................    3      1    JCK-3
....................................................................................................................................................................................................................uuccacagcCuucuugaacu.....................    3      1    JCK-2
```

```
                                                                                                                                                                                                  Star                                                          Mature
ucucuuucucucucucucucucuaaaaaaaacaaaaggaaagaaaaaaaaaaaaaaaaaacuuacuacuacuacuaguaguauaucuucuaauuugcuggaaaggcacaccaaagagaaagaaauuaaaccaaggaaggaaggaaggaaggaaggaagugagaucugaugagagaaaaagagaauguuuuugcaaguccuggucaugcuuuuccacagcuuucuugaacuuucuuucuuucuguuuagucu
..................................................................................................................................................................................................................uuccacagcuuucuugaacC.....................    18     1    LT-3
..................................................................................................................................................................................................................uuccacagcuuucuugaacA.....................    20     1    JT-1
..................................................................................................................................................................................................................uuccacagcCuucuugaacu.....................    7      1    LT-2
..................................................................................................................................................................................................................uuccacagUuuucuugaacu.....................    1      1    JCK-2
..................................................................................................................................................................................................................uuccacagcuuucuugaacu.....................    2316   0    LCK-1
..................................................................................................................................................................................................................uuccacagcuuucuugaacC.....................    39     1    LT-2
..................................................................................................................................................................................................................uuccacaAcuuucuugaacu.....................    1      1    LT-1
..................................................................................................................................................................................................................uuccacagcuuucCugaacu.....................    1      1    JCK-2
..................................................................................................................................................................................................................uuccaAagcuuucuugaacu.....................    7      1    LCK-3
..................................................................................................................................................................................................................uuccacagcuuucuugaacA.....................    43     1    JCK-3
..................................................................................................................................................................................................................uuccacagcuuucuugCacu.....................    3      1    JCK-2
..................................................................................................................................................................................................................uuccacagcuuuAuugaacu.....................    2      1    JCK-3
..................................................................................................................................................................................................................uuccacagcuuucuCgaacu.....................    1      1    JCK-1
..................................................................................................................................................................................................................uuccacagcuuucuugaCcu.....................    3      1    JT-1
..................................................................................................................................................................................................................uuccacagcuuucNugaacu.....................    5      1    LCK-1
..................................................................................................................................................................................................................uuccacagcuCucuugaacu.....................    2      1    LT-2
..................................................................................................................................................................................................................uuccacaAcuuucuugaacu.....................    4      1    LCK-3
..................................................................................................................................................................................................................uuccacagcuuucuugaacA.....................    57     1    JCK-2
..................................................................................................................................................................................................................uuccacagcuuucuugaacA.....................    172    1    LT-3
..................................................................................................................................................................................................................uuccacagcuuucuugaCcu.....................    3      1    JCK-1
..................................................................................................................................................................................................................uuccacagcuuucuugaaAu.....................    6      1    LCK-3
..................................................................................................................................................................................................................uuccacagcuuucuugaacu.....................    6887   0    LT-2
..................................................................................................................................................................................................................uuccacagcuuucuugaacC.....................    41     1    LCK-3
..................................................................................................................................................................................................................uuccacagcuuucuugCacu.....................    10     1    LT-2
..................................................................................................................................................................................................................uuccacagcuuCcuugaacu.....................    2      1    LT-2
..................................................................................................................................................................................................................uuccCcagcuuucuugaacu.....................    3      1    LT-1
..................................................................................................................................................................................................................uuccacagcuuucuuUaacu.....................    1      1    LT-2
..................................................................................................................................................................................................................uuccacagcuuucuugaacA.....................    87     1    LT-1
..................................................................................................................................................................................................................uuccacaUcuuucuugaacu.....................    1      1    JCK-1
..................................................................................................................................................................................................................uuccacagcuuuNuugaacu.....................    1      1    LCK-1
..................................................................................................................................................................................................................uuccacCgcuuucuugaacu.....................    9      1    LCK-3
..................................................................................................................................................................................................................uuccCcagcuuucuugaacu.....................    2      1    JT-1
..................................................................................................................................................................................................................uuccacagcuuucuugaacA.....................    3      1    JT-2
..................................................................................................................................................................................................................uuccacagcuuucuugaCcu.....................    3      1    LCK-1
..................................................................................................................................................................................................................Cuccacagcuuucuugaacu.....................    1      1    LT-2
..................................................................................................................................................................................................................uucUacagcuuucuugaacu.....................    3      1    LCK-3
..................................................................................................................................................................................................................uuccacagcuuucuugaaUu.....................    111    1    JCK-2
..................................................................................................................................................................................................................uuccacagcuuucuugaNcu.....................    2      1    JCK-3
..................................................................................................................................................................................................................uCccacagcuuucuugaacu.....................    1      1    LT-1
..................................................................................................................................................................................................................uuccacagcuuGcuugaacu.....................    1      1    JCK-1
..................................................................................................................................................................................................................uCccacagcuuucuugaacu.....................    1      1    LT-3
..................................................................................................................................................................................................................uuccacagcuuucuugaacC.....................    3      1    JT-3
..................................................................................................................................................................................................................uuccacagcuuucuuAaacu.....................    1      1    LT-2
..................................................................................................................................................................................................................uuccacCgcuuucuugaacu.....................    3      1    JCK-3
..................................................................................................................................................................................................................uuccacagcuuucuugaGcu.....................    3      1    JCK-1
..................................................................................................................................................................................................................uuccacagcuuucuugaacC.....................    9      1    LT-1
..................................................................................................................................................................................................................uuccacagcuuucuugaacC.....................    2      1    LCK-1
..................................................................................................................................................................................................................uuccacagcuuucuugCacu.....................    10     1    LCK-3
..................................................................................................................................................................................................................uuccacagcuuuAuugaacu.....................    2      1    LT-3
..................................................................................................................................................................................................................uuccacCgcuuucuugaacu.....................    2      1    LCK-1
..................................................................................................................................................................................................................uuGcacagcuuucuugaacu.....................    5      1    LT-2
..................................................................................................................................................................................................................uuccCcagcuuucuugaacu.....................    2      1    JT-3
..................................................................................................................................................................................................................Cuccacagcuuucuugaacu.....................    2      1    JCK-3
..................................................................................................................................................................................................................uuccacCgcuuucuugaacu.....................    8      1    LT-3
..................................................................................................................................................................................................................uuccacagcuuucuugaaUu.....................    24     1    LCK-1
..................................................................................................................................................................................................................uNccacagcuuucuugaacu.....................    1      1    JT-1
..................................................................................................................................................................................................................uuccacagcuuucuugaaUu.....................    132    1    LT-1
..................................................................................................................................................................................................................uuccCcagcuuucuugaacu.....................    20     1    LT-2
..................................................................................................................................................................................................................uuccacCgcuuucuugaacu.....................    6      1    JCK-2
..................................................................................................................................................................................................................uuccacCgcuuucuugaacu.....................    13     1    LT-2
..................................................................................................................................................................................................................uuccacagcuuucuugaaAu.....................    1      1    JCK-1
..................................................................................................................................................................................................................uuccacagcuuNcuugaacu.....................    1      1    JT-2
..................................................................................................................................................................................................................uuccacagcuuucuugaacG.....................    2      1    LT-1
..................................................................................................................................................................................................................uuccacagcuuucuugaacG.....................    2      1    JCK-2
..................................................................................................................................................................................................................uuccaAagcuuucuugaacu.....................    1      1    JCK-2
..................................................................................................................................................................................................................uuccacagcuuucuugaaUu.....................    52     1    JCK-3
..................................................................................................................................................................................................................uuccacagcuuucuugaacG.....................    8      1    LT-2
..................................................................................................................................................................................................................uuccacagcuuucuugaacA.....................    196    1    LCK-3
..................................................................................................................................................................................................................uuccaAagcuuucuugaacu.....................    3      1    LT-3
..................................................................................................................................................................................................................Cuccacagcuuucuugaacu.....................    1      1    JCK-2
```

Star: ggaaggaaggaagugagau
Mature: uuccacagcuuucuugaacu

ucucuuucucucucucucucucuaaaaaaaacaaaaggaaagaaaaaaaaaaaaaaaaaacuuacuacuacuacuaguaguauaucuucuaauuugcuggaaaggcacaccaaagagaaagaaauuaaaccaaggaaggaaggaaggaaggaaggaagugagaucugaugagagaaaaagagaauguuuuugcaaguccuggucaugcuuuuccacagcuuucuugaacuuucuuucuuucuguuuagucu

uuccacagcCuucuugaacu	5	1	LT-1
uuccacagcuuucuugaCcu	1	1	LT-1
uuccacagcuuucuNgaacu	7	1	LCK-1
uuccacagcuuucuugaCcu	1	1	LCK-2
uuccacagcuuucGugaacu	1	1	LCK-1
uuccacagcuuucuugaaAu	6	1	LT-2
uuccacagcuuucuugaaUu	72	1	JCK-1
uuccacagcCuucuugaacu	1	1	LT-3
uuccacagcuuucuugaCcu	8	1	LT-3
uuccacagcuuucuugGacu	8	1	LCK-3
uuccacagcuuucuugaaUu	172	1	LT-3
uuccacagcuuucuugaaUu	18	1	JT-1
uuccacagcuuucuugCacu	2	1	LCK-1
uuccacagcuuuAuugaacu	4	1	LCK-3
uuccCcagcuuucuugaacu	8	1	LCK-3
uuccCcagcuuucuugaacu	4	1	JCK-1
uuccacagcuuucGugaacu	1	1	LCK-2
uuccaUagcuuucuugaacu	3	1	JCK-2
uuccacagcuuucuugaacC	7	1	JCK-1
uucUacagcuuucuugaacu	1	1	JCK-1
uuccacagcuuucuugaGcu	2	1	LCK-3
uuccacGgcuuucuugaacu	1	1	LCK-3
uuccacagcuuucuugaacA	2	1	LCK-1
uuccacaAcuuucuugaacu	1	1	JT-1
uuUcacagcuuucuugaacu	4	1	JCK-2
uuccacagcuCucuugaacu	1	1	JT-1
uuccacagcuuucuugaUcu	2	1	LT-1
uuccacagcuuucuugCacu	2	1	JCK-3
uuccacagcuuucuuCaacu	1	1	JCK-1
uuccacagcuuucuugaacu	4415	0	LT-1
uuccacagcuuCcuugaacu	1	1	JCK-2
uuccacagcuuucuugaaUu	35	1	LCK-2
uuccCcagcuuucuugaacu	1	1	LCK-1
uCccacagcuuucuugaacu	1	1	LCK-3
uuccacagcuuucuugaCcu	3	1	JCK-2
uuccacagcuuucuGgaacu	1	1	LT-3
Cuccacagcuuucuugaacu	2	1	LCK-1
uuccacagcuuucuugaacG	1	1	JT-1
uuccacagcuuucuugaacu	2326	0	LCK-2
uNccacagcuuucuugaacu	2	1	JCK-3
uuccacagcuuucuugaacA	137	1	LT-2
uuccacagcCuucuugaacu	1	1	JT-2
uNccacagcuuucuugaacu	4	1	JCK-1
Cuccacagcuuucuugaacu	2	1	LT-1
uuccaNagcuuucuugaacu	3	1	LCK-1
Cuccacagcuuucuugaacu	1	1	LT-3
uuNcacagcuuucuugaacu	33	1	LT-2
uucUacagcuuucuugaacu	3	1	LT-2
uuccacaAcuuucuugaacu	1	1	LT-3
uuccacaAcuuucuugaacu	1	1	LT-2
uuccacagcuuucuugaacG	6	1	LT-3
uuccacagcuuucCugaacu	2	1	LCK-3
uuccacagcuuucCugaacu	1	1	LT-3
uuccacagcuuNcuugaacu	3	1	JCK-1
uucNacagcuuucuugaacu	2	1	JCK-1
uuccacagcuuucuugCacu	2	1	LCK-2
Cuccacagcuuucuugaacu	4	1	LCK-3
uuccacagcuuucuugaacuC	33	1	LT-1
uuccCcagcuuucuugaacuu	6	1	JCK-2
uuccacagcuuucuCgaacuu	5	1	LT-2
uuccacagcuuuUuugaacuu	1	1	JCK-2
uuccacagcuuuAuugaacuu	1	1	LT-3
uuccacagcuuucuugaacuC	9	1	LCK-2
uuccacagcuuucuugaacuC	68	1	JCK-3
uucNacagcuuucuugaacuu	2	1	JCK-1
uuccacagcuuucuugaacuA	75	1	JCK-1
uuccacagcuuucuugaacuu	1769	0	LT-3
uuccacagcuuucuugaacuA	97	1	JT-1
uuccaAagcuuucuugaacuu	3	1	JCK-3
uuccacagcuuucuugaacuA	148	1	LT-3

```
                                                                                                                                                                                                        Star                                                Mature
ucucuuucucucucucucucucucuaaaaaaacaaaaggaaagaaaaaaaaaaaaaaaaacuuacuacuacuacuaguaguauaucuucuaauuugcuggaaaggcacaccaaagagaaagaaauuaaaccaaggaaggaaggaaggaaggaaggaagugagaucugaugagagaaaaagagaauguuuuugcaaguccuggucaugcuuuuccacagcuuucuugaacuuucuuucuuucuguuuagucu
........................................................................................................................................................................................................uCccacagcuuucuugaacuu....................    1     1    JCK-2
........................................................................................................................................................................................................uuccacagcuuucuugaacGu....................    1     1    JCK-2
........................................................................................................................................................................................................uuccacagcuuucuugCacuu....................    2     1    LT-2
........................................................................................................................................................................................................uuccCcagcuuucuugaacuu....................    2     1    JCK-1
........................................................................................................................................................................................................uuccCcagcuuucuugaacuu....................    2     1    JCK-3
........................................................................................................................................................................................................uuccacagcuuucuugaCcuu....................    1     1    LT-3
........................................................................................................................................................................................................uuccaAagcuuucuugaacuu....................    1     1    LT-2
........................................................................................................................................................................................................uCccacagcuuucuugaacuu....................    1     1    LCK-2
........................................................................................................................................................................................................uuccacagcuuucuugaacuA....................    2     1    JT-2
........................................................................................................................................................................................................uuccacCgcuuucuugaacuu....................    1     1    LCK-2
........................................................................................................................................................................................................uuccaAagcuuucuugaacuu....................    2     1    JCK-1
........................................................................................................................................................................................................uNccacagcuuucuugaacuu....................    6     1    JCK-3
........................................................................................................................................................................................................uuccacagcuuucuugaacGu....................    1     1    LT-3
........................................................................................................................................................................................................uuccacagcuuucCugaacuu....................    1     1    JCK-1
........................................................................................................................................................................................................uuccacCgcuuucuugaacuu....................    3     1    JCK-3
........................................................................................................................................................................................................uNccacagcuuucuugaacuu....................    1     1    LT-2
........................................................................................................................................................................................................uuccacagcuuucuugaacAu....................    6     1    LT-3
........................................................................................................................................................................................................uuccaAagcuuucuugaacuu....................    5     1    JCK-2
........................................................................................................................................................................................................uuccacagcuuucuugCacuu....................    2     1    JCK-3
........................................................................................................................................................................................................uuccacagcuuucuugaacuu....................    2427  0    LCK-3
........................................................................................................................................................................................................uuccCcagcuuucuugaacuu....................    1     1    LCK-3
........................................................................................................................................................................................................uuccacagcuuucuugCacuu....................    2     1    LT-3
........................................................................................................................................................................................................uuccaNagcuuucuugaacuu....................    2     1    LCK-1
........................................................................................................................................................................................................uuccacagcuuucuugaaUuu....................    101   1    LT-2
........................................................................................................................................................................................................uuccacagcuuucuugaaAuu....................    5     1    LT-2
........................................................................................................................................................................................................uuccacagcuuucuugaacuu....................    2955  0    JCK-3
........................................................................................................................................................................................................uuccacagcuuucuugaacuC....................    6     1    LCK-1
........................................................................................................................................................................................................uuccacagcuuucuugaacuA....................    66    1    LT-1
........................................................................................................................................................................................................uuccacagcuuucuuAaacuu....................    1     1    JCK-1
........................................................................................................................................................................................................uuccacaAcuuucuugaacuu....................    1     1    JCK-3
........................................................................................................................................................................................................uuccacagcuuucuugaaUuu....................    29    1    LCK-1
........................................................................................................................................................................................................uuccacagcuuucuugaacAu....................    3     1    JCK-3
........................................................................................................................................................................................................uuccacagcuuucuugaacuu....................    3888  0    JCK-2
........................................................................................................................................................................................................uuccacCgcuuucuugaacuu....................    5     1    JCK-1
........................................................................................................................................................................................................uuccacagcuuucCugaacuu....................    1     1    LT-3
........................................................................................................................................................................................................uuccacagcuuucCugaacuu....................    1     1    JCK-2
........................................................................................................................................................................................................uNccacagcuuucuugaacuu....................    1     1    JCK-1
........................................................................................................................................................................................................uuccacagcuuucuugaacuu....................    1431  0    LT-2
........................................................................................................................................................................................................uuccacCgcuuucuugaacuu....................    3     1    LT-3
........................................................................................................................................................................................................uuccacaAcuuucuugaacuu....................    1     1    LT-2
........................................................................................................................................................................................................uuccacagcuuucuugaacuA....................    240   1    LCK-3
........................................................................................................................................................................................................uuccacagcuuucuugaaAuu....................    3     1    LCK-3
........................................................................................................................................................................................................uuccCcagcuuucuugaacuu....................    3     1    LT-3
........................................................................................................................................................................................................uuccacCgcuuucuugaacuu....................    3     1    LCK-3
........................................................................................................................................................................................................uuccacagcuuucuugaacNu....................    2     1    JCK-1
........................................................................................................................................................................................................Cuccacagcuuucuugaacuu....................    3     1    JCK-3
........................................................................................................................................................................................................uuccacagcuuucuugaacuu....................    954   0    LT-1
........................................................................................................................................................................................................uuccacagcuuucuugaaUuu....................    11    1    JT-3
........................................................................................................................................................................................................uuUcacagcuuucuugaacuu....................    6     1    JCK-2
........................................................................................................................................................................................................uuccacagcuuuAuugaacuu....................    1     1    LCK-3
........................................................................................................................................................................................................uuccacagcuuucuugaCcuu....................    3     1    LCK-3
........................................................................................................................................................................................................uuccacagcuuucuugaacuC....................    53    1    LCK-3
........................................................................................................................................................................................................uuccacagcuuucuugaacuN....................    4     1    JCK-1
........................................................................................................................................................................................................uuccacagcuuucuuAaacuu....................    1     1    LT-1
........................................................................................................................................................................................................uuccacGgcuuucuugaacuu....................    1     1    JCK-2
........................................................................................................................................................................................................uuccacagcuuucuugaCcuu....................    2     1    JCK-3
........................................................................................................................................................................................................uuccaUagcuuucuugaacuu....................    3     1    JCK-3
........................................................................................................................................................................................................uucNacagcuuucuugaacuu....................    1     1    JT-1
........................................................................................................................................................................................................uuccacagcuuucuuAaacuu....................    1     1    LCK-2
........................................................................................................................................................................................................uuUcacagcuuucuugaacuu....................    2     1    JCK-3
........................................................................................................................................................................................................uuccacagcuuCcuugaacuu....................    1     1    LT-2
........................................................................................................................................................................................................uuccacagcuuucuugaaAuu....................    1     1    LT-1
........................................................................................................................................................................................................uuccacagcuuucuugaaUuu....................    50    1    JCK-1
........................................................................................................................................................................................................uuccacagcuuucuugaacuA....................    23    1    LCK-2
........................................................................................................................................................................................................uuccacagcuuucuugaacCu....................    1     1    LT-2
........................................................................................................................................................................................................uuccacagcuuucuugCacuu....................    1     1    LCK-3
........................................................................................................................................................................................................uuccacagcuuucuugaaUuu....................    48    1    LCK-2
........................................................................................................................................................................................................uuccacagcuuucuugaacuA....................    20    1    LCK-1
........................................................................................................................................................................................................uuccacGgcuuucuugaacuu....................    1     1    JCK-1
........................................................................................................................................................................................................uuccacagcuuucuugaacuC....................    44    1    LT-2
```

Star — ggaaggaaggaagugagau

Mature — uuccacagcuuucuugaacu

```
ucucuuucucucucucucucucucuaaaaaaacaaaaggaaagaaaaaaaaaaaaaaaaacuuacuacuacuacuaguaguauaucuucuaauuugcuggaaaggcacaccaaagagaaagaaauuaaaccaaggaaggaaggaaggaaggaaggaagugagaucugaugagagaaaaagagaauguuuuugcaaguccuggucaugcuuuuccacagcuuucuugaacuuucuuucuuucuguuuagucu
```

uuccacCgcuuucuugaacuu	3	1	JCK-2
uuUcacagcuuucuugaacuu	1	1	JCK-1
uuccacagcuuucuugaacAu	6	1	LT-2
uuccacCgcuuucuugaacuu	1	1	JT-1
uuccacagcuuucuugaCcuu	3	1	JCK-1
uuccacagNuuucuugaacuu	1	1	JCK-3
uCccacagcuuucuugaacuu	1	1	LT-2
uuccacagcuuucuugaacuC	54	1	LT-3
uuccacagcuuucuugaacuA	134	1	LT-2
uuUcacagcuuucuugaacuu	1	1	JT-3
uuccacagcuuucuugaacuu	157	0	LCK-1
uuccacagcuuucuugaacuC	53	1	JCK-1
uuccacagcuuuUuugaacuu	1	1	LT-1
uuccacagcuuucuugaCcuu	5	1	JCK-2
uuccacagcuuucuugaacuu	191	0	LCK-2
uuccacagcuuucuugaacAu	8	1	LCK-3
uNccacagcuuucuugaacuu	4	1	JCK-2
uuccacagcuuucuugCacuu	1	1	JT-1
uuccacagcuuNcuugaacuu	3	1	JCK-1
uuccacagcuuucuugaacuA	67	1	JCK-3
uuccacagcuuuAuugaacuu	1	1	LT-2
uuccacGgcuuucuugaacuu	11	1	JT-3
uuccacagcuuucuugaaUuu	3	1	JT-2
uuccacagcuuucuugaaUuu	61	1	JCK-3
uuccacGgcuuucuugaacuu	7	1	JT-2
uuccacagcuuucuugaacuu	502	0	JT-1
uuccaUagcuuucuugaacuu	1	1	LT-3
uuccaUagcuuucuugaacuu	1	1	JCK-1
uuccacagcuuucuuAaacuu	2	1	LCK-3
uucGacagcuuucuugaacuu	1	1	JCK-1
uuccacaAcuuucuugaacuu	1	1	LT-1
uuccCcagcuuucuugaacuu	5	1	LT-2
uCccacagcuuucuugaacuu	1	1	LCK-3
uuccacagcuuucuugaacuu	2349	0	JCK-1
uuccacagcuuucuugCacuu	1	1	LT-1
uuccacagcCuucuugaacuu	1	1	JCK-3
uuccacagcuuucuugaacuA	5	1	JT-3
uuccacagcuuucuugaacuC	18	1	JT-1
uuccacagcuuucuugaacuC	64	1	JCK-2
uuccacagcuuucuugaacAu	2	1	JCK-1
uuccacagcuuucuugaaUuu	112	1	LCK-3
uuccacagcuuucuugaCcuu	4	1	LT-2
uucUacagcuuucuugaacuu	1	1	JCK-3
uuccacagAuuucuugaacuu	1	1	JCK-1
uuccacagcuuucuugaaUuu	88	1	JCK-2
uuccacagcuuucuugCacuu	2	1	JCK-1
uuUcacagcuuucuugaacuu	2	1	JT-2
uuccacagcuuucuugaacuA	125	1	JCK-2
uuccacagcuCucuugaacuu	1	1	JT-2
uuccacagcuuucuugCacuu	5	1	JCK-2
uuccacagcuuucuugaacAu	1	1	LT-1
uuccacagcuuuAuugaacuu	3	1	JCK-3
uuccacagcuuucuugaCcuu	2	1	JT-1
uuccacagcuuucuugaacAu	5	1	JCK-2
uuccacaAcuuucuugaacuu	1	1	LCK-3
uNccacagcuuucuugaacuu	1	1	JT-1
uuccacagcCuucuugaacuu	2	1	LT-1
uuccacagcuuucuugaaUuu	86	1	LT-1
uucUacagcuuucuugaacuu	4	1	JCK-2
uuccacagcuuucuugaacuu	97	0	JT-2
uuccacagcuuucuugaacuu	145	0	JT-3
uuccacagcuuucuugaaUuu	135	1	LT-3
uuccacagcuuucuugaacGu	1	1	LT-1
uuccacagcuuucuugaGcuu	4	1	JCK-1
uucNacagcuuucuugaacuu	1	1	JCK-3
uuNcacagcuuucuugaacuu	4	1	LT-2
Cuccacagcuuucuugaacuu	1	1	LT-3
uuccacagcuuucuugaacuuC	8	1	JT-2
uuccacagcuuucuuUaacuuu	1	1	LT-3
uuccacagcuuucuugaacCuu	1	1	LCK-1

Star Mature

```
ucucuuucucucucucucucuaaaaaaaacaaaaggaaagaaaaaaaaaaaaaaaaaaacuuacuacuacuacuaguaguauaucuucuaauuugcuggaaaggcacaccaaagagaaagaaauuaaaccaaggaaggaaggaaggaaggaaggaagugagaucugaugagagaaaaagagaauguuuuugcaaguccuggucaugcuuuuccacagcuuucuugaacuuucuuucuuucuguuuagucu
.................................................................................................................................................................................................................uuccacagcuuucuugaacuuC...................   73    1   LT-1
.................................................................................................................................................................................................................uuccacCgcuuucuugaacuuu...................   8     1   LT-2
.................................................................................................................................................................................................................uuccacagcuuucuugaacuuG...................   2     1   JCK-2
.................................................................................................................................................................................................................uuccacagcuuucNugaacuuu...................   1     1   LCK-1
.................................................................................................................................................................................................................uuccacaAcuuucuugaacuuu...................   1     1   JT-3
.................................................................................................................................................................................................................uuccacagcCuucuugaacuuu...................   2     1   LT-2
.................................................................................................................................................................................................................uuccacagcuuucuugaacuuC...................   253   1   LCK-3
.................................................................................................................................................................................................................uuGcacagcuuucuugaacuuu...................   2     1   LT-2
.................................................................................................................................................................................................................uuccacagcuuucuugaaUuuu...................   3     1   JT-2
.................................................................................................................................................................................................................uNccacagcuuucuugaacuuu...................   1     1   JCK-3
.................................................................................................................................................................................................................uuccacagcuuucuugaaUuuu...................   7     1   JT-1
.................................................................................................................................................................................................................uuccacCgcuuucuugaacuuu...................   2     1   LT-1
.................................................................................................................................................................................................................uuccacagcuuucuugaaUuuu...................   32    1   LT-3
.................................................................................................................................................................................................................uuccacagcuuucuugaacuuA...................   128   1   JCK-2
.................................................................................................................................................................................................................uuccacagcuuucuugaaAuuu...................   2     1   LT-3
.................................................................................................................................................................................................................uuccacagcuuucuugaacuCu...................   2     1   JCK-1
.................................................................................................................................................................................................................Cuccacagcuuucuugaacuuu...................   1     1   JT-1
.................................................................................................................................................................................................................uuccacagcuuucuugaacAuu...................   29    1   LT-3
.................................................................................................................................................................................................................uuccacagcuuucuugaacuuA...................   157   1   LCK-3
.................................................................................................................................................................................................................uuccacagcuuucuugaacuuG...................   2     1   LT-1
.................................................................................................................................................................................................................uuccacagcuuucuCgaacuuu...................   3     1   LT-3
.................................................................................................................................................................................................................uuccacagcuuucuugaCcuuu...................   3     1   LT-2
.................................................................................................................................................................................................................uuccacagcuuucuugaaUuuu...................   1     1   JT-3
.................................................................................................................................................................................................................uuccacagcuuucuugCacuuu...................   1     1   JCK-1
.................................................................................................................................................................................................................uucUacagcuuucuugaacuuu...................   1     1   LCK-3
.................................................................................................................................................................................................................uuccaAagcuuucuugaacuuu...................   1     1   JCK-2
.................................................................................................................................................................................................................uuccacagcuuucuugaacuuA...................   77    1   JCK-3
.................................................................................................................................................................................................................uuccCcagcuuucuugaacuuu...................   1     1   JCK-3
.................................................................................................................................................................................................................uuccacCgcuuucuugaacuuu...................   1     1   JCK-1
.................................................................................................................................................................................................................uuccacagcuuucuugaacuuA...................   50    1   JCK-1
.................................................................................................................................................................................................................uuUcacagcuuucuugaacuuu...................   7     1   LT-2
.................................................................................................................................................................................................................uuccacagcCuucuugaacuuu...................   2     1   JCK-1
.................................................................................................................................................................................................................uuccCcagcuuucuugaacuuu...................   2     1   JCK-2
.................................................................................................................................................................................................................uuccacagcuuucuugaacuAu...................   3     1   JCK-1
.................................................................................................................................................................................................................uuccacagcuuucuugaaUuuu...................   37    1   LT-2
.................................................................................................................................................................................................................uuccacagcuuucuugaacuuA...................   121   1   LT-2
.................................................................................................................................................................................................................uuUcacagcuuucuugaacuuu...................   1     1   JCK-1
.................................................................................................................................................................................................................uuccacagcuuucuugaacuuA...................   21    1   LCK-1
.................................................................................................................................................................................................................uuccacagcuuucCugaacuuu...................   1     1   JCK-2
.................................................................................................................................................................................................................uuccacagcCuucuugaacuuu...................   2     1   JCK-2
.................................................................................................................................................................................................................uuccacCgcuuucuugaacuuu...................   1     1   JT-1
.................................................................................................................................................................................................................uuccacagcuuucuugaacGuu...................   1     1   LT-3
.................................................................................................................................................................................................................uuccacagcuuucuugaaUuuu...................   39    1   LCK-3
.................................................................................................................................................................................................................uuNcacagcuuucuugaacuuu...................   4     1   LT-2
.................................................................................................................................................................................................................uuccacagcuuucuugaacuCu...................   6     1   JCK-2
.................................................................................................................................................................................................................uuccacagcuuucuugaacuuC...................   114   1   LT-2
.................................................................................................................................................................................................................uCccacagcuuucuugaacuuu...................   1     1   LT-1
.................................................................................................................................................................................................................uuccacagcuuucuugaacGuu...................   8     1   JCK-2
.................................................................................................................................................................................................................uuccacagcuuucuugaacuuN...................   1     1   JCK-1
.................................................................................................................................................................................................................uuUcacagcuuucuugaacuuu...................   1     1   LT-3
.................................................................................................................................................................................................................uuccacagcuuucuugaacCuu...................   3     1   JCK-3
.................................................................................................................................................................................................................uuccacagcuuucuugaaAuuu...................   4     1   JCK-1
.................................................................................................................................................................................................................uuccacagcuuucuugaaUuuu...................   41    1   LT-1
.................................................................................................................................................................................................................uuccacagcuuucuugaacuuC...................   41    1   JT-1
.................................................................................................................................................................................................................uuccacagcuuucuugaacuNu...................   1     1   JT-2
.................................................................................................................................................................................................................uNccacagcuuucuugaacuuu...................   1     1   JCK-2
.................................................................................................................................................................................................................uuccacagcuuucuugaacuAu...................   13    1   LT-2
.................................................................................................................................................................................................................uuccacagcuuucuugaacuAu...................   6     1   JCK-3
.................................................................................................................................................................................................................uuccacagcuuucuugaacuuu...................   1108  0   JCK-3
.................................................................................................................................................................................................................uuccacagcuuucuugaacAuu...................   26    1   LT-2
.................................................................................................................................................................................................................uuccacagcuuucuugaCcuuu...................   1     1   LCK-2
.................................................................................................................................................................................................................uuccacagcuuucuugaacuuA...................   121   1   LT-3
.................................................................................................................................................................................................................uuccaUagcuuucuugaacuuu...................   1     1   LT-1
.................................................................................................................................................................................................................uuccacagcuuucuugaacuuu...................   246   0   LCK-2
.................................................................................................................................................................................................................uuccacagcuuucuugaacAuu...................   12    1   JCK-3
.................................................................................................................................................................................................................uuccCcagcuuucuugaacuuu...................   4     1   LT-3
.................................................................................................................................................................................................................uuccacagcuuucuugaacuAu...................   11    1   LT-1
.................................................................................................................................................................................................................uuccacagcuuucuugaacuuu...................   1068  0   LT-1
.................................................................................................................................................................................................................uuccacagcuuucuugaacuuG...................   7     1   LT-2
.................................................................................................................................................................................................................uuccacCgcuuucuugaacuuu...................   3     1   JCK-3
```

Star — **Mature**

ucucuuucucucucucucucucuaaaaaaacaaaaggaaagaaaaaaaaaaaaaaaaaacuuacuacuacuacuaguaguauaucuucuaauuugcuggaaaggcacaccaaagagaaagaaauuaaaccaaggaaggaaggaaggaaggaaggaagugagaucugaugagagaaaaagagaauguuuuugcaaguccuggucaugcuuuuccacagcuuucuugaacuuucuuucuuucuguuuagucu

uuccacagcuuucuugaacuuu	64	0	JT-3
uuccacCgcuuucuugaacuuu	1	1	LCK-3
uuccacagcuuucuugaacCuu	1	1	LT-3
uuccacagcuuucuugaacAuu	12	1	LT-1
Cuccacagcuuucuugaacuuu	1	1	JCK-3
uuccacagcuuuAuugaacuuu	4	1	LT-2
uuccacagcuuucuugaacuuC	89	1	JCK-2
uuccacagcuuucuugaacAuu	14	1	JCK-1
uuccacagcuuucuugaCcuuu	1	1	LCK-3
uuccGcagcuuucuugaacuuu	1	1	LT-2
uuccacagcuuucuugaacuuu	250	0	LCK-1
uuccacagcuuucuugaCcuuu	2	1	LT-3
uuccacagcuuCcuugaacuuu	1	1	LT-2
Cuccacagcuuucuugaacuuu	1	1	JCK-1
uuccacCgcuuucuugaacuuu	2	1	LT-3
uuccacagcuuucuugaacuuA	17	1	JT-3
uuccacagcuuucuugaacuuu	2105	0	LCK-3
uuccacagcuuucuugCacuuu	1	1	JCK-3
uuccacagcuuucuugCacuuu	1	1	JCK-2
uuccacagcuuucuugaacuAu	17	1	LCK-3
uuccCcagcuuucuugaacuuu	1	1	JCK-1
uuccacagcuuucuugaacAuu	36	1	JCK-2
uNccacagcuuucuugaacuuu	2	1	LCK-3
uuccacagcuuucuugCacuuu	2	1	LT-2
uuccacagcuuucuugCacuuu	1	1	JT-3
uuccacagcuuucuugaacAuu	4	1	JT-3
uuccacagcuuucuugaacuuA	49	1	JT-1
uuccacagcuuucuugaacuNu	1	1	JCK-1
uuccacagcuuucuugaacuuu	70	0	JT-2
uuccacaAcuuucuugaacuuu	2	1	LT-2
uuccacagcuuucuugaacuuA	10	1	JT-2
uuccacagcuuucuugaacuAu	16	1	JCK-2
uuccacagcuuucuugaCcuuu	2	1	JCK-2
uuccacagcuuucuugCacuuu	1	1	LCK-3
uuccacagcuuGcuugaacuuu	2	1	JCK-1
uuccacagcuuucuugaaUuuu	27	1	JCK-1
uuccacCgcuuucuugaacuuu	2	1	JCK-2
uuccacagcuuucuugaacuCu	3	1	LT-3
uuccacagcuGucuugaacuuu	2	1	JCK-1
uuccacagcuuuAuugaacuuu	1	1	LT-1
uuccacagcuuucuugCacuuu	1	1	LCK-2
uuccacagcuuucuugaacuCu	1	1	LT-2
uuccacagcuuuAuugaacuuu	2	1	JCK-1
uuccacagcuuucuugaacCuu	3	1	JCK-2
uuccacagcCuucuugaacuuu	4	1	JCK-3
Cuccacagcuuucuugaacuuu	1	1	LT-3
uuccacagcuuucuugaacuCu	5	1	LCK-3
uuccCcagcuuucuugaacuuu	4	1	LCK-3
uuccacagcuuucuugaacuuC	55	1	JCK-3
uuccacagcuuucuugaacuuu	899	0	JCK-1
uuccacagcuuucuugaacuuu	1432	0	LT-2
uuccacagcuuucuugaacuuu	1512	0	LT-3
uuccacagcuuucuugaaAuuu	2	1	JT-3
uuccacCgcuuucuugaacuuu	2	1	LCK-2
uuccacagcuuucuugaaUuuu	36	1	JCK-3
uucNacagcuuucuugaacuuu	1	1	JCK-1
uCccacagcuuucuugaacuuu	3	1	LT-3
uuccacagcuuucuugaacuuG	3	1	LT-3
uuccacagcuuucuugaacuuN	1	1	LT-2
uuccacagcuuucuugaacuuC	12	1	JT-3
uuccaAagcuuucuugaacuuu	1	1	JCK-1
uuccacagcuuucuugaacuAu	12	1	LT-3
uuccacagcuCucuugaacuuu	1	1	LT-1
uuccacagcuuucuugaCcuuu	2	1	LT-1
uuccaAagcuuucuugaacuuu	1	1	JCK-3
uuccCcagcuuucuugaacuuu	3	1	LT-2
uuccCcagcuuucuugaacuuu	1	1	LT-1
uuccacagcuuucuugaacGuu	3	1	JCK-1
uuccacagcuuucuugaaUuuu	22	1	LCK-1
uuccacagcuuucuugaacuuu	377	0	JT-1

Star Mature

ucucuuucucucucucucucucuaaaaaaaacaaaaggaaagaaaaaaaaaaaaaaaaacuuacuacuacuacuaguaguauaucuucuaauuugcuggaaaggcacaccaaagagaaagaaauuaaaccaaggaaggaaggaaggaaggaaggaagugagaucugaugagagaaaaagagaauguuuuugcaaguccuggucaugcuuuuccacagcuuucuugaacuuucuuucuuucuguuuagucu

uuccacagcuuucuugaacAuu	6	1	JT-2
uuccacagcuuucuugaacuCu	1	1	LT-1
uuccaNagcuuucuugaacuuu	1	1	LCK-1
uCccacagcuuucuugaacuuu	1	1	JCK-3
uuccacagcuuNcuugaacuuu	1	1	JT-1
uuccacagcuuucuugaaAuuu	3	1	JCK-2
uuccacagcuuucuugCacuuu	2	1	LT-1
uuccacagcuuucuugaacAuu	16	1	LCK-3
uuccacagcuuucuugaacuuA	31	1	LCK-2
uuccacagcuuucuugaaAuuu	2	1	JCK-3
uuUcacagcuuucuugaacuuu	1	1	JCK-2
uuccacagcuuucuugaaUuuu	13	1	LCK-2
uGccacagcuuucuugaacuuu	1	1	LCK-3
uNccacagcuuucuugaacuuu	1	1	JCK-1
uuccacagcuuucuugaacCuu	1	1	JCK-1
uuccacagcuuucuugaacuuu	1496	0	JCK-2
uuccacagcuuNcuugaacuuu	3	1	JCK-1
uuccacagcuGucuugaacuuu	1	1	LT-2
uuccacagcuuucuugaacuuC	118	1	LT-3
uuccacagcuuucuugaacuCu	5	1	JCK-3
uuccacagcuuucuugaacuuA	62	1	LT-1
uuccacagcuuucuugaacuuC	65	1	JCK-1
uuccacagcuuucuugaaUuuu	63	1	JCK-2
uuccacagcuuucuugaacuuAc	5	1	JT-3
uuccacagcuuucuugaacAuuc	1	1	JCK-1
uuccacagcuuucuugaacuuuG	9	1	LCK-3
uuccacagcuuucuugaaUuuuc	3	1	JCK-2
uuccacagcuuucuugaacAuuc	1	1	JCK-3
uuccacagcuuucuugCacuuuc	1	1	LCK-3
uuccacagcuuucuugaaUuuuc	6	1	LCK-3
uuccacagcuuucuugaacuuuG	11	1	LT-3
uuccacagcuuucuugaacuuAc	1	1	JT-2
uuccacagcuuucuugaacuuuG	9	1	LT-2
uuccacagcuuucuugaacuuuG	4	1	JCK-1
uuccacagcuuucuugaacuuuU	492	1	JCK-2
uuccacagcuuucuugaacuuuU	9	1	JT-2
uuccacagcuuucuugaacuuCc	1	1	LT-1
uuccacagcuuucuugaaUuuuc	15	1	LCK-1
uuccCcagcuuucuugaacuuuc	1	1	LT-2
uuccacagcuuucuugaacuuuG	4	1	JCK-3
uuccacagcuuucuugaacuuuA	11	1	JT-2
uuccacagcuuucuugaaAuuuc	1	1	LT-3
uuccacagcuuucuugCacuuuc	1	1	LT-2
uuccacagcuuucuugaacuAuc	7	1	LT-2
uuccacagcuuucuugaacuuuA	144	1	JCK-1
uuccacagcuuucuugaacAuuc	10	1	LCK-3
uuccacagcuuucuugaacAuuc	4	1	LT-1
uuccacagcuuucuugaacuuuc	40	0	JCK-3
uuccacagcuuucuugaacAuuc	2	1	LT-2
uuccacagcuuucuugaacuuuA	166	1	LT-3
uuccacagcuuucuugaacuuuG	13	1	LT-1
uuccacagcuuucuugaacuuuc	3	0	JT-3
uuccacagcuuucuugaacuuuU	294	1	JCK-1
uuccacagcuuucuugaacuuuA	40	1	LCK-1
uuccacagcuuucuugaacuuuc	33	0	JCK-1
uuccacagcuuucuugaacAuuc	3	1	LT-3
uuccacagcuuucuugaacuuuG	8	1	JCK-2
uuccacagcuuucuugaacuAuc	1	1	LT-3
uuccacagcuuucuugaacuuuU	490	1	LCK-3
uuccacagcuuucuugaacuuuA	47	1	JT-1
uuccacagcuuucuugaacuuuU	306	1	LT-1
uuccacagcuuucuugaacuuuU	134	1	LCK-2
uuccacCgcuuucuugaacuuuc	1	1	LCK-3
uuccacagcuuucuugaacuuuA	48	1	LCK-2
uuccacagcuuucuugaacuuuA	119	1	LT-1
uuccacagcuuucuugaacuuuU	140	1	LCK-1
uuccacagcuuucuugaaUuuuc	4	1	LT-1
uuccacagcuuucuugaacuuuA	155	1	JCK-3
uuccacagcuuucuugaacuuuU	369	1	JCK-3
uuccacagcuuucuugaacuuuA	230	1	LT-2

```
                                                                                                                                                                                                                  Star                                                                                         Mature
ucucuuucucucucucucucucucuaaaaaaaacaaaaggaaagaaaaaaaaaaaaaaaaacuuacuacuacuacuaguaguauaucuucuaauuugcuggaaaggcacaccaaagagaaagaaauuaaaccaaggaaggaaggaaggaaggaaggaagugagaucugaugagagaaaaagagaauguuuuugcaaguccuggucaugcuuuuccacagcuuucuugaacuuucuuucuuucuguuuagucu
................................................................................................................................................................................................................uuccacagcuuucuugaacuuuc..................   3     0   JT-2
................................................................................................................................................................................................................uuccacagcuuucuugaaUuuuc..................   5     1   JCK-3
................................................................................................................................................................................................................uuccacagcuuucuugaacuAuc..................   4     1   LT-1
................................................................................................................................................................................................................uuccacagcuuucuugaacuuAc..................   1     1   LT-2
................................................................................................................................................................................................................uuccacagcuuucuugaacuuuU..................   101   1   JT-1
................................................................................................................................................................................................................uuccacagcuuucuugaacuuuc..................   47    0   LT-3
................................................................................................................................................................................................................uuccacagcuuucuugaaUuuuc..................   13    1   LCK-2
................................................................................................................................................................................................................uuccacGgcuuucuugaacuuuc..................   1     1   LT-2
................................................................................................................................................................................................................uuccacagcuuucuugaacuuuc..................   28    0   LT-1
................................................................................................................................................................................................................uuccacCgcuuucuugaacuuuc..................   1     1   LT-2
................................................................................................................................................................................................................uuccacagcuuucuugaacuuuc..................   61    0   LT-2
................................................................................................................................................................................................................uuccacagcuuucuugaacuuuU..................   18    1   JT-3
................................................................................................................................................................................................................uuccacagcuuucuugaacuuuU..................   376   1   LT-2
................................................................................................................................................................................................................uuccacagcuuucuugaacuuuc..................   16    0   JT-1
................................................................................................................................................................................................................uuccacagcuuucuugaacuuuc..................   97    0   LCK-3
................................................................................................................................................................................................................uuccacagcuuucuugaacuuuU..................   324   1   LT-3
................................................................................................................................................................................................................uuccacagcuuucuugaacuuAc..................   1     1   LT-3
................................................................................................................................................................................................................uuccacagcuuucuugaacuuuA..................   301   1   LCK-3
................................................................................................................................................................................................................uuccacagcuuucuugaacuuAc..................   1     1   JCK-3
................................................................................................................................................................................................................uuccacagcuuucuugaacuuuA..................   177   1   JCK-2
................................................................................................................................................................................................................uuccacagcuuucuugaacuuuc..................   50    0   JCK-2
................................................................................................................................................................................................................uuccacagcuuucuugaacuuuA..................   16    1   JT-3
................................................................................................................................................................................................................uuNcacagcuuucuugaacuuuc..................   1     1   LT-2
................................................................................................................................................................................................................uuccacagcuuucuugaacuuuAu.................   2     1   JCK-1
................................................................................................................................................................................................................uuccacagcuuucuugaacuuucA.................   2     1   JCK-2
................................................................................................................................................................................................................uuccacagcuuucuugaacuuAcu.................   1     1   LT-3
................................................................................................................................................................................................................uuccacagcuuucuugaacuAucu.................   4     1   JCK-2
................................................................................................................................................................................................................uuccacagcuuucuugaacuuuUu.................   19    1   LT-1
................................................................................................................................................................................................................uuccacagcuuucuugaacuuucA.................   6     1   LT-2
................................................................................................................................................................................................................uuccacagcuuucuugaacuuucA.................   1     1   LT-1
................................................................................................................................................................................................................uuccacagcuuucuugaacuuuAu.................   2     1   LT-1
................................................................................................................................................................................................................uuccacagcuuucuugaacuuuAu.................   5     1   LCK-3
................................................................................................................................................................................................................uuccacagcuuucuugaacuuucA.................   6     1   JT-1
................................................................................................................................................................................................................uuccacagcuuucuugaacuuAcu.................   2     1   LT-2
................................................................................................................................................................................................................uuccacagcuuucuugaacuAucu.................   1     1   JCK-1
................................................................................................................................................................................................................uuccacagcuuucuugaacuuuAu.................   7     1   JCK-3
................................................................................................................................................................................................................uuccacagcuuucuugaacuuuUu.................   13    1   LCK-3
................................................................................................................................................................................................................uuccacagcuuucuugaacuuuUu.................   15    1   LT-2
................................................................................................................................................................................................................uuccacagcuuucuugaacuuucA.................   1     1   LT-3
................................................................................................................................................................................................................uuccacagcuuucuugaacuuuAu.................   4     1   JT-1
................................................................................................................................................................................................................uuccacagcuuucuugaacuAucu.................   5     1   LCK-3
................................................................................................................................................................................................................uuccacagcuuucuugaacuuAcu.................   2     1   JCK-2
................................................................................................................................................................................................................uuccacagcuuucuugaacuuucu.................   2     0   LCK-3
................................................................................................................................................................................................................uuccacagcuuucuugaacuuAcu.................   1     1   LT-1
................................................................................................................................................................................................................uuccacagcuuucuugaacuuuAu.................   6     1   JCK-2
................................................................................................................................................................................................................uuccacagcuuucuugaacuuucA.................   1     1   JCK-1
................................................................................................................................................................................................................uuccacagcuuucuugaacuuuAu.................   6     1   LT-3
................................................................................................................................................................................................................uuccacagcuuucuugaacuuuUu.................   47    1   JCK-1
................................................................................................................................................................................................................uuccacagcuuucuugaacuuuAu.................   2     1   LT-2
................................................................................................................................................................................................................uuccacagcuuucuugaacuuuUu.................   49    1   JCK-3
................................................................................................................................................................................................................uuccacagcuuucuugaacuuuUu.................   89    1   JCK-2
................................................................................................................................................................................................................uuccacagcuuucuugaacuuucu.................   3     0   LT-1
................................................................................................................................................................................................................uuccacagcuuucuugaacuuuUu.................   13    1   LT-3
................................................................................................................................................................................................................uuccacagcuuucuugaacuuucA.................   4     1   JCK-3
................................................................................................................................................................................................................uuccacagcuuucuugaacuAucu.................   1     1   JCK-3
................................................................................................................................................................................................................uuccacagcuuucuugaacuuCcu.................   1     1   LCK-3
................................................................................................................................................................................................................uuccacagcuuucuugaacuuuUuu................   2     1   LT-3
................................................................................................................................................................................................................uuccacagcuuucuugaacuuucuA................   1     1   LT-2
................................................................................................................................................................................................................uuccacagcuuucuugaacuuuAuu................   7     1   LCK-3
................................................................................................................................................................................................................uuccacagcuuucuugaacuuuUuu................   12    1   JCK-2
................................................................................................................................................................................................................uuccacagcuuucuugaacuuuUuu................   9     1   JCK-1
................................................................................................................................................................................................................uuccacagcuuucuugaacuuuUuu................   5     1   LT-2
................................................................................................................................................................................................................uuccacagcuuucuugaacuuuUuu................   1     1   LT-1
................................................................................................................................................................................................................uuccacagcuuucuugaacuuuAuu................   8     1   JCK-2
................................................................................................................................................................................................................uuccacagcuuucuugaacuuuAuu................   3     1   LT-3
................................................................................................................................................................................................................uuccacagcuuucuugaacuuuAuu................   4     1   LT-2
................................................................................................................................................................................................................uuccacagcuuucuugaacuuucuC................   1     1   LT-3
................................................................................................................................................................................................................uuccacagcuuucuugaacuuuGuu................   1     1   LT-3
................................................................................................................................................................................................................uuccacagcuuucuugaacuuuAuu................   12    1   JCK-3
................................................................................................................................................................................................................uuccacagcuuucuugaacuuuUuu................   2     1   LCK-3
```

```
Star                                                                                                                                                                                                                         Mature
ucucuuucucucucucucucucucuaaaaaaaacaaaaggaaagaaaaaaaaaaaaaaaaaaacuuacuacuacuacuaguaguauaucuucuaauuugcuggaaaggcacaccaaagagaaagaaauuaaaccaaggaaggaaggaaggaaggaaggaagugagaucugaugagagaaaaagagaauguuuuugcaaguccuggucaugcuuuuccacagcuuucuugaacuuucuuucuuucuguuuagucu
....................................................................................................................................................................................................................uuccacagcuuucuugaacuuAcuu................	3	1	JCK-2
....................................................................................................................................................................................................................uuccacagcuuucuugaacuuuAuu...............	5	1	LT-1
....................................................................................................................................................................................................................uuccacagcuuucuugaacuuuAuu...............	7	1	JCK-1
....................................................................................................................................................................................................................uuccacagcuuucuugaacuuuUuu...............	6	1	JCK-3
....................................................................................................................................................................................................................uuccacagcuuucuugaacuuuUuuu..............	2	1	LCK-3
....................................................................................................................................................................................................................uuccacagcuuucuugaacuuuAuuu..............	5	1	JCK-3
....................................................................................................................................................................................................................uuccacagcuuucuugaacuuuAuuu..............	3	1	LCK-3
....................................................................................................................................................................................................................uuccacagcuuucuugaacuuuAuuu..............	3	1	JCK-2
....................................................................................................................................................................................................................uuccacagcuuucuugaacuuuAuuu..............	3	1	JCK-1
....................................................................................................................................................................................................................uuccacagcuuucuugaacuuucuuu..............	1	0	JCK-1
.....................................................................................................................................................................................................................ucNacagcuuucuugaac.....................	1	1	LT-2
.....................................................................................................................................................................................................................uccacaUcuuucuugaac.....................	1	1	LT-3
.....................................................................................................................................................................................................................uccacagcuuucuugaac.....................	10	0	LT-2
.....................................................................................................................................................................................................................uccacagcuuucuugaac.....................	2	0	JCK-2
.....................................................................................................................................................................................................................Cccacagcuuucuugaac.....................	1	1	LT-1
.....................................................................................................................................................................................................................uccacagcuuucuugaac.....................	11	0	LT-3
.....................................................................................................................................................................................................................uccacagcuuucuugaac.....................	2	0	JCK-3
.....................................................................................................................................................................................................................uccacagcuuucuugaac.....................	2	0	JT-2
.....................................................................................................................................................................................................................uccacagcuuucuugaac.....................	13	0	LCK-3
.....................................................................................................................................................................................................................uccacagcuuucuugaac.....................	6	0	JT-1
.....................................................................................................................................................................................................................uccacagcuuucuugaac.....................	4	0	JT-3
.....................................................................................................................................................................................................................uccacagcuuucuugaac.....................	4	0	LT-1
.....................................................................................................................................................................................................................uccacagcuCucuugaac.....................	1	1	LT-3
.....................................................................................................................................................................................................................uccacagcuuucuugaacA....................	1	1	LT-1
.....................................................................................................................................................................................................................uccacagcuuucuugaacu....................	7	0	LT-1
.....................................................................................................................................................................................................................uccacagcuuucuugaaUu....................	1	1	LT-1
.....................................................................................................................................................................................................................uccacagcuuucuugaacA....................	1	1	LT-3
.....................................................................................................................................................................................................................uccacagcuuucuugaacu....................	53	0	LCK-1
.....................................................................................................................................................................................................................uccacagcuuucuugaacu....................	2	0	JCK-1
.....................................................................................................................................................................................................................uccacagcuuucuugaacu....................	12	0	LCK-3
.....................................................................................................................................................................................................................uccacagcuuucuugaacu....................	7	0	JCK-3
.....................................................................................................................................................................................................................uccacagcuuucuugaacu....................	10	0	JCK-2
.....................................................................................................................................................................................................................uccacagcuuucuugaacu....................	29	0	LCK-2
.....................................................................................................................................................................................................................uccacagcuuucuugaaAu....................	3	1	LT-2
.....................................................................................................................................................................................................................uccacagcuuucuugaacu....................	18	0	LT-3
.....................................................................................................................................................................................................................uccacagcuuucuugaacu....................	2	0	LT-2
.....................................................................................................................................................................................................................uccacagcuuucuugaacuu...................	5	0	JCK-3
.....................................................................................................................................................................................................................uccacagcuuucuugaacuu...................	6	0	JCK-2
.....................................................................................................................................................................................................................uccacagcuuucuugaacuu...................	2	0	LT-1
.....................................................................................................................................................................................................................uccacagcuuucuugaacuu...................	2	0	LT-3
.....................................................................................................................................................................................................................uccacagcuuucuugaaUuu...................	1	1	LT-3
.....................................................................................................................................................................................................................uccacagcuuucuugaacuu...................	9	0	LCK-3
.....................................................................................................................................................................................................................uccacagcuuucuugaacuu...................	7	0	LT-2
.....................................................................................................................................................................................................................uccacagcuuucuugaacuu...................	1	0	JCK-1
.....................................................................................................................................................................................................................uccacagcuuucuugaacAuu..................	4	1	LT-2
.....................................................................................................................................................................................................................uccacagcuuucuugaacuuu..................	2	0	JCK-1
.....................................................................................................................................................................................................................uccacagcuuucuugaacuuu..................	2	0	LT-1
.....................................................................................................................................................................................................................Cccacagcuuucuugaacuuu..................	1	1	JCK-1
.....................................................................................................................................................................................................................uccacagcuuucuugaacuuu..................	4	0	LCK-3
.....................................................................................................................................................................................................................uccacagcuuucuugaacuuu..................	1	0	JCK-2
.....................................................................................................................................................................................................................uccacagcuuucuugaacuuC..................	1	1	LT-2
.....................................................................................................................................................................................................................uccacagcuuucuugaacuuu..................	5	0	LT-2
.....................................................................................................................................................................................................................uccacagcuuucuugaacuuu..................	4	0	LT-3
.....................................................................................................................................................................................................................uccacagcuuucuugaacuuuc.................	1	0	JCK-1
.....................................................................................................................................................................................................................uccacagcuuucuugaacuuuU.................	2	1	LT-3
.....................................................................................................................................................................................................................uccacagcuuucuugaacuuuU.................	6	1	LCK-3
.....................................................................................................................................................................................................................uccacagcuuucuugaacuuuU.................	2	1	JCK-3
.....................................................................................................................................................................................................................uccacagcuuucuugaacuuuU.................	1	1	LT-2
.....................................................................................................................................................................................................................uccacagcuuucuugaacuuuc.................	2	0	LT-1
.....................................................................................................................................................................................................................uccacagcuuucuugaacuuuU.................	3	1	LT-1
.....................................................................................................................................................................................................................uccacagcuuucuugaacuuuA.................	1	1	LT-2
.....................................................................................................................................................................................................................uccacagcuuucuugaacuuuA.................	10	1	LCK-3
.....................................................................................................................................................................................................................uccacagcuuucuugaacuuuU.................	1	1	JCK-1
.....................................................................................................................................................................................................................uccacagcuuucuugaacuuuU.................	3	1	JCK-2
.....................................................................................................................................................................................................................uccacagcuuucuugaacuuuc.................	1	0	JCK-2
.....................................................................................................................................................................................................................uccacagcuuucuugaacuuuUu................	2	1	LCK-3
......................................................................................................................................................................................................................ccacagcuuucuugaacC....................	2	1	JCK-1
......................................................................................................................................................................................................................ccacagcuuucuugaacu....................	36	0	LCK-2
......................................................................................................................................................................................................................ccacagcuuucuugaacu....................	342	0	LT-3
......................................................................................................................................................................................................................ccacagcuuucuugaacC....................	1	1	LT-3
```

Star Mature

ucucuuucucucucucucucucucuaaaaaaacaaaaggaaagaaaaaaaaaaaaaaaaacuuacuacuacuacuaguaguauaucuucuaauuugcuggaaaggcacaccaaagagaaagaaauuaaaccaaggaaggaaggaaggaaggaaggaagugagaucugaugagagaaaaagagaauguuuuugcaaguccuggucaugcuuuuccacagcuuucuugaacuuucuuucuuucuguuuagucu

ccacagcuuucuugaacu	301	0	LT-2
ccacagcuuucuugaacu	51	0	JCK-1
ccacagcuuucuugaacG	1	1	LT-3
ccacagcuuucuugaacA	1	1	JCK-2
ccacagcuuucuugaaUu	2	1	LCK-3
ccacagcuuucuugaacu	51	0	JT-1
ccacagcuuucuugaCcu	2	1	LCK-3
ccacagcuuucuugaaUu	5	1	LT-3
ccacagcuuuUuugaacu	4	1	LT-3
ccacagcuuucuugaacA	1	1	LT-3
Acacagcuuucuugaacu	1	1	LT-2
ccacagcuuucuugaacu	318	0	LCK-3
ccacagcuuuAuugaacu	1	1	LT-3
ccacagcuuucuugaacu	104	0	LT-1
ccacagcuuucuugaacu	89	0	JCK-2
ccacagcuuucuugaCcu	1	1	LT-3
ccacagcuuucuugaCcu	1	1	JT-1
ccacagcuuucuugaacu	14	0	JT-2
ccacagcuuucuugaacA	2	1	LT-2
ccCcagcuuucuugaacu	1	1	LT-2
ccacagcuuucuugaaUu	2	1	LT-1
ccacCgcuuucuugaacu	1	1	LT-3
ccacagcuuucuugaacA	1	1	JCK-1
ccacagcuuucuugCacu	2	1	LT-3
ccacagcCuucuugaacu	1	1	LT-3
ccacagcuuucuugaacu	20	0	JT-3
ccacagcuuucuugaacu	24	0	LCK-1
ccacagcuuucuugaacu	69	0	JCK-3
ccacagcuuucuugaacuA	9	1	LT-3
ccCcagcuuucuugaacuu	1	1	LT-3
ccacagcuuucuugaacuu	68	0	JT-1
ccacagcuuucuugCacuu	2	1	JCK-3
ccacCgcuuucuugaacuu	1	1	JT-1
ccacagcuuucuugaacuC	10	1	JT-1
ccacagcuuucuugaacuu	251	0	LT-3
ccacagcuuucuugaCcuu	1	1	JT-2
ccacagcuuucuugaaUuu	4	1	JCK-3
ccacagcuuucuugaacuA	17	1	JCK-2
ccaAagcuuucuugaacuu	1	1	JCK-1
cNacagcuuucuugaacuu	1	1	JCK-3
ccacCgcuuucuugaacuu	1	1	JCK-2
ccacagcuuucuugaacuA	19	1	LT-2
ccacagcCuucuugaacuu	3	1	LCK-3
ccacagcuuucuugaacuA	10	1	JT-1
ccacagcuuucuugaaUuu	9	1	LT-2
ccacagcuuucuugaacAu	1	1	JCK-1
ccacagcuuucuugaacuA	41	1	LCK-3
ccacagcuuucuugaacuA	3	1	JT-2
ccacagcuuucuugaCcuu	1	1	LCK-3
ccacagcuuucuugaaUuu	5	1	JCK-2
cNacagcuuucuugaacuu	1	1	JCK-2
ccacagcuuucuugaacuu	252	0	JCK-2
ccacagcuuucuugaaAuu	3	1	LT-3
ccacagcuuucuugaaUuu	3	1	JCK-1
cNacagcuuucuugaacuu	1	1	JT-1
ccacagcuuucuugaacuC	12	1	JCK-2
ccacagcuuucuugaacuu	246	0	LT-2
ccacagcuuucuugaacuC	4	1	JCK-3
ccacagcuuucuugaaUuu	6	1	LCK-3
ccacagcuuucuugaacuA	11	1	JCK-1
ccacagcuuucuugaacCu	1	1	LCK-3
ccacagcuuucuugaacuu	42	0	JT-3
ccacagcuuucuugaacuC	3	1	LT-2
ccacagcuuucuugaacuA	5	1	JT-3
ccacagcuuucuugaaUuu	3	1	LT-1
ccacagcuuucuugCacuu	1	1	JCK-2
ccacagcuuucuugaacuu	180	0	JCK-3
ccacagcuuucuugaacuC	2	1	JCK-1
ccacCgcuuucuugaacuu	2	1	LCK-3
ccacagcuuucuugaacuu	34	0	JT-2

Star Mature

ucucuuucucucucucucucucucuaaaaaaaacaaaaggaaagaaaaaaaaaaaaaaaaacuuacuacuacuacuaguaguauaucuucuaauuugcuggaaaggcacaccaaagagaaagaaauuaaaccaaggaaggaaggaaggaaggaaggaagugagaucugaugagagaaaaagagaauguuuuugcaaguccuggucaugcuuuuccacagcuuucuugaacuuucuuucuuucuguuuagucu

...ccacagcuuucuugaacuA...	6	1	LT-1
...ccacagcuuucuugCacuu...	2	1	LT-2
...ccacagcuuucuugaacuC...	5	1	LT-1
...ccacagcuuucuugaacuu...	168	0	JCK-1
...ccacagcuuucuugaacuu...	349	0	LCK-3
...ccacagcuuucuugaacuC...	4	1	LT-3
...ccacagcuuucuugaaUuu...	7	1	LT-3
...ccacagcuuucuugaacuA...	8	1	JCK-3
...ccacagcuuucuugaaAuu...	1	1	JCK-2
...ccaNagcuuucuugaacuu...	1	1	JCK-1
...ccacagcuuucuugaCcuu...	1	1	LT-2
...ccacagcuuucNugaacuu...	1	1	JCK-1
...ccacagcuuucuugaacuu...	66	0	LT-1
...ccacagcuuucuugaacuuu...	506	0	LCK-3
...ccacagcuuucuugaacuCu...	7	1	JCK-3
...ccacagcuuucuugaacAuu...	1	1	JCK-2
...ccacagcuuucuugCacuuu...	1	1	LCK-3
...ccacagcuuucuugaacuAu...	16	1	LT-2
...ccacagcuuucuugaacuuA...	53	1	JCK-3
...ccacagcuuucuugaacuuu...	21	0	JT-2
...ccacagcuuucuugaacuCu...	4	1	LT-1
...ccacagcuuucuugaCcuuu...	1	1	JCK-3
...ccaAagcuuucuugaacuuu...	1	1	LCK-1
...ccacagcuuucuugaacAuu...	2	1	LT-3
...ccacagcuuucuugaacuuA...	10	1	LT-1
...ccacagcuuucuugaaUuuu...	13	1	LCK-3
...ccacagcuuucuugaaUuuu...	27	1	LCK-1
...ccacCgcuuucuugaacuuu...	2	1	LT-2
...ccacagcuuucuugaaUuuu...	3	1	JCK-3
...ccacagcuuucuugaacuuC...	5	1	LCK-3
...ccacagcuuucuugaacuuA...	40	1	LCK-3
...ccacagcuuucuugaacuuu...	31	0	JT-3
...ccacagcuuucuugaacuuu...	71	0	LCK-2
...ccacagcuuucuugaacAuu...	2	1	LT-1
...Ucacagcuuucuugaacuuu...	1	1	JCK-1
...ccacagcuuucuugaacuuG...	1	1	JCK-3
...ccacagcuuucuugaacuuG...	1	1	LT-3
...ccacagcuuucuugaaAuuu...	5	1	LCK-3
...ccacagcuuucuugaacuuC...	20	1	LT-3
...ccacagcuuucuugaacuCu...	8	1	LT-3
...ccacCgcuuucuugaacuuu...	1	1	JCK-1
...Ucacagcuuucuugaacuuu...	4	1	JCK-2
...ccacagcuuucuugaacuCu...	4	1	JCK-1
...ccacagcuuucuugaacuuA...	41	1	JCK-1
...ccacagcuuucuugaacuuu...	77	0	LCK-1
...ccacagcuuucuugaacuAu...	9	1	JCK-2
...ccacagcCuucuugaacuuu...	1	1	JCK-3
...ccacagcuuucuuAaacuuu...	1	1	LT-2
...ccacagcuuucuugaacuAu...	6	1	JCK-1
...ccacagcuuucuugaacuuA...	48	1	LT-3
...ccacagcuuucuugaacuCu...	17	1	JCK-2
...ccacagcuuucuugaacAuu...	5	1	LCK-3
...ccacagcuuucuugaacuuG...	2	1	JCK-2
...ccacagcuuucuugaacuAu...	9	1	LT-3
...ccacagcuuucuugaacuuu...	495	0	JCK-2
...cNacagcuuucuugaacuuu...	1	1	JCK-1
...ccacagcuuucuugNacuuu...	1	1	LCK-1
...ccacagcuuucuugaacuuA...	8	1	JT-3
...ccacagcuuucuugaacuuA...	51	1	LT-2
...ccacagcuuucuugaacuuu...	485	0	LT-2
...ccacagcuuucuugaacuuu...	169	0	LT-1
...ccacagcuuucuugaaUuuu...	4	1	JCK-1
...ccacagcuuucuugaacAuu...	3	1	LT-2
...ccacaAcuuucuugaacuuu...	1	1	JCK-3
...ccacagcuuucuugaacuuC...	4	1	LT-1
...ccacagcuuucuugaacuuG...	7	1	JCK-1
...ccacagcuuucuugCacuuu...	1	1	LT-2
...ccacagcuuucuugaGcuuu...	1	1	LCK-2
...ccacagcuuucuugaacuuu...	43	0	JT-1
...ccNcagcuuucuugaacuuu...	1	1	LCK-1

Star Mature

ucucuuucucucucucucucucuaaaaaaaacaaaaggaaagaaaaaaaaaaaaaaaaacuuacuacuacuacuaguaguauaucuucuaauuugcuggaaaggcacaccaaagagaaagaaauuaaaccaaggaaggaaggaaggaaggaaggaagugagaucugaugagagaaaaagagaauguuuuugcaaguccuggucaugcuuuuccacagcuuucuugaacuuucuuucuuucuguuuagucu

ccacCgcuuucuugaacuuu	3	1	JCK-3
ccacagcuuucuugaacuuC	14	1	LT-2
ccacCgcuuucuugaacuuu	1	1	JT-1
ccacagcuuucuugCacuuu	1	1	JCK-2
ccacagcuuucuugaacuuC	24	1	JCK-2
ccacagcuuucuugaacuuG	1	1	LT-1
ccacagcuuucuugaacuuC	15	1	JCK-1
ccacagcuuucuugaacuuC	18	1	JCK-3
ccacagcuuucuugaacAuu	2	1	JCK-1
ccacaNcuuucuugaacuuu	1	1	LCK-1
ccacagcuuucuugaacuCu	4	1	LT-2
ccacagcuuucuugaacGuu	1	1	JCK-1
ccacagcuuucuugaaUuuu	7	1	LT-3
ccacagcuuucuugaacuuu	334	0	JCK-1
ccacagcuuCcuugaacuuu	1	1	LT-3
ccacagcuuucuugaacuuu	349	0	JCK-3
ccacagcuuucuugaacuAu	9	1	LCK-3
ccacagcuuucuugaaUuuu	18	1	LCK-2
ccacagGuuucuugaacuuu	1	1	JT-3
ccacagcuuucuugaacuuA	11	1	JT-2
ccacagcuuucuugaacuuA	69	1	JCK-2
ccacagcuuucuugaacuuu	439	0	LT-3
ccacagcuuucuugaacuAu	5	1	JCK-3
ccacagcuuucuugaacuuuc	30	0	LCK-2
ccacagcuuucuugaacuuuU	349	1	LCK-3
ccacagcuuucuugaacuuuc	29	0	LCK-3
ccacagcuuucuugaacuuuG	6	1	LT-1
ccacagcuuucuugaacuuuG	8	1	JCK-2
ccacagcuuucuugaacuuuU	223	1	LT-3
ccacagcuuucuugaacuuuG	10	1	JCK-3
ccacagcuuucuugaacuuuA	146	1	LT-2
ccacagcuuucuugaacuuuU	13	1	JT-3
ccacagcuuucuugaacuuuU	153	1	JCK-3
ccacagcuuucuugaaUuuuc	1	1	LT-2
ccacagcuuucuugaacuuAc	1	1	JCK-2
ccacagcuuucuugaacuuuU	87	1	LT-1
ccacagcuuucuugaacuuuc	27	0	LCK-1
ccacagcuuucuugaacuuuc	3	0	LT-1
ccacagcuuucuugaacuuuA	150	1	JCK-1
ccacagcuuucuugaacuuuU	246	1	LT-2
ccacagcuuucuugaacuuuU	2	1	JT-2
ccacagcuuucuugaCcuuuc	1	1	LT-1
ccacagcuuucuugaacuuuG	6	1	LT-3
ccacagcuuucuugaacuuuU	52	1	LCK-1
ccacagcuuucuugaacuuuA	90	1	JCK-3
ccacagcuuucuugaacuuCc	1	1	LCK-3
ccacagcuuucuugaacuuAc	1	1	LT-2
ccacagcuuucuugaacuuuc	8	0	LT-3
ccacagcuuucuugaacuuuU	64	1	LCK-2
ccacagcuuucuugaacuuCc	1	1	JCK-1
ccacagcuuucuugaacuAuc	1	1	LT-3
ccacagcuuucuugaacuuuG	9	1	JCK-1
ccacagcuuucuugaacuuuU	42	1	JT-1
ccacagcuuucuugaacuuuc	8	0	JCK-2
ccacagcuuucuugaacuuuc	16	0	LT-2
ccacagcuuucuugaacuuAc	3	1	LT-3
ccacagcuuucuugaacuuuA	109	1	LCK-3
ccacagcuuucuugaacuuuA	82	1	LT-3
ccacagcuuucuugaacuuuc	9	0	JCK-3
ccacagcuuucuugaacuuuG	17	1	LCK-3
ccacagcuuucuugaacuuuA	7	1	JT-1
ccacagcuuucuugaacuuuA	33	1	LT-1
ccacagcuuucuugaacuuuA	178	1	JCK-2
ccacagcuuucuugaacuuCc	1	1	LT-3
ccacagcuuucuugaacuuAc	5	1	LCK-3
ccacagcuuucuugaacuuuG	14	1	LT-2
ccacagcuuucuugaacuuuc	4	0	JCK-1
ccacagcuuucuugaacuuuU	138	1	JCK-1
ccacagcuuucuugaacuuuU	184	1	JCK-2
ccacagcuuucuugaacuAuc	7	1	LCK-3

Star Mature

```
ucucuuucucucucucucucucucuaaaaaaaacaaaaggaaagaaaaaaaaaaaaaaaaaacuuacuacuacuacuaguaguauaucuucuaauuugcuggaaaggcacaccaaagagaaagaaauuaaaccaaggaaggaaggaaggaaggaaggaagugagaucugaugagagaaaaagagaauguuuuugcaaguccuggucaugcuuuuccacagcuuucuugaacuuucuuucuuucuguuuagucu
......................................................................................................................................................................................................................ccacagcuuucuugaacuuuUu.................   29   1   JCK-1
......................................................................................................................................................................................................................ccacagcuuucuugaacuuuUu.................   2    1   JT-2
......................................................................................................................................................................................................................ccacagcuuucuugaacuuAcu.................   1    1   LT-1
......................................................................................................................................................................................................................ccacagcuuucuugaacuuuAu.................   1    1   JT-3
......................................................................................................................................................................................................................ccacagcuuucuugaacuuucu.................   1    0   JCK-1
......................................................................................................................................................................................................................ccacagcuuucuugaacuuAcu.................   23   1   LCK-3
......................................................................................................................................................................................................................ccacagcuuucuugaacuuucu.................   4    0   JCK-3
......................................................................................................................................................................................................................ccacagcuuucuugaacuuucC.................   2    1   LT-3
......................................................................................................................................................................................................................ccacagcuuucuugaacuuucu.................   1    0   LT-1
......................................................................................................................................................................................................................ccacagcuuucuugaacuuuAu.................   16   1   JCK-3
......................................................................................................................................................................................................................ccacagcuuucuugaacuuuUu.................   38   1   JCK-3
......................................................................................................................................................................................................................ccacagcuuucuugaacuuuAu.................   16   1   LT-2
......................................................................................................................................................................................................................ccacagcuuucuugaacuuAcu.................   1    1   JCK-1
......................................................................................................................................................................................................................ccacagcuuucuugaacuuuUu.................   54   1   JCK-2
......................................................................................................................................................................................................................ccacagcuuucuugaacuuuAu.................   23   1   LCK-3
......................................................................................................................................................................................................................ccacagcuuucuugaacuuuAu.................   4    1   LT-1
......................................................................................................................................................................................................................ccacagcuuucuugaacuuucA.................   3    1   LT-3
......................................................................................................................................................................................................................ccacagcuuucuugaacuuAcu.................   4    1   JCK-2
......................................................................................................................................................................................................................ccacagcuuucuugaacuuuUu.................   4    1   JT-3
......................................................................................................................................................................................................................ccacagcuuucuugaacuuAcu.................   2    1   LT-3
......................................................................................................................................................................................................................ccacagcuuucuugaacuuucG.................   5    1   JCK-2
......................................................................................................................................................................................................................ccacagcuuucuugaacuuuUu.................   22   1   LCK-3
......................................................................................................................................................................................................................ccacagcuuucuugaacuuuGu.................   5    1   LCK-3
......................................................................................................................................................................................................................ccacagcuuucuugaacuuAcu.................   3    1   LT-2
......................................................................................................................................................................................................................ccacagcuuucuugaacuuucA.................   4    1   LT-2
......................................................................................................................................................................................................................ccacagcuuucuugaacuuucu.................   3    0   LT-3
......................................................................................................................................................................................................................ccacagcuuucuugaacuuucA.................   1    1   JCK-1
......................................................................................................................................................................................................................ccacagcuuucuugaacuuuAu.................   13   1   LT-3
......................................................................................................................................................................................................................ccacagcuuucuugaacuuucG.................   1    1   JCK-3
......................................................................................................................................................................................................................ccacagcuuucuugaacuuuUu.................   25   1   LT-3
......................................................................................................................................................................................................................ccacagcuuucuugaacuuAcu.................   4    1   JCK-3
......................................................................................................................................................................................................................ccacagcuuucuugaacuuucu.................   4    0   JCK-2
......................................................................................................................................................................................................................ccacagcuuucuugaacuuuGu.................   3    1   LT-1
......................................................................................................................................................................................................................ccacagcuuucuugaacuuuUu.................   39   1   LT-2
......................................................................................................................................................................................................................ccacagcuuucuugaacuuuUu.................   7    1   LT-1
......................................................................................................................................................................................................................ccacagcuuucuugaacuuuAu.................   23   1   JCK-2
......................................................................................................................................................................................................................ccacagcuuucuugaacuuucA.................   5    1   JCK-2
......................................................................................................................................................................................................................ccacagcuuucuugaacuAucu.................   2    1   LCK-3
......................................................................................................................................................................................................................ccacagcuuucuugaacuuuAu.................   19   1   JCK-1
......................................................................................................................................................................................................................ccacagcuuucuugaacuuucA.................   3    1   JCK-3
......................................................................................................................................................................................................................ccacagcuuucuugaacuuuUuu................   11   1   JCK-2
......................................................................................................................................................................................................................ccacagcuuucuugaacuuucuA................   2    1   LCK-3
......................................................................................................................................................................................................................ccacagcuuucuugaacuuuUuu................   5    1   LT-2
......................................................................................................................................................................................................................ccacagcuuucuugaacuuuAuu................   3    1   LT-1
......................................................................................................................................................................................................................ccacagcuuucuugaacuuuUuu................   5    1   LT-1
......................................................................................................................................................................................................................ccacagcuuucuugaacuuucuA................   1    1   LT-2
......................................................................................................................................................................................................................ccacagcuuucuugaacuuuAuu................   4    1   JT-3
......................................................................................................................................................................................................................ccacagcuuucuugaacuuuAuu................   13   1   LT-2
......................................................................................................................................................................................................................ccacagcuuucuugaacuuucAu................   1    1   JCK-2
......................................................................................................................................................................................................................ccacagcuuucuugaacuuuUuu................   4    1   JCK-1
......................................................................................................................................................................................................................ccacagcuuucuugaacuuuAuu................   4    1   LT-3
......................................................................................................................................................................................................................ccacagcuuucuugaacuuuAuu................   25   1   LCK-3
......................................................................................................................................................................................................................ccacagcuuucuugaacuuuGuu................   1    1   JCK-2
......................................................................................................................................................................................................................ccacagcuuucuugaacuuuUuu................   14   1   LCK-3
......................................................................................................................................................................................................................ccacagcuuucuugaacuuucCu................   1    1   JCK-2
......................................................................................................................................................................................................................ccacagcuuucuugaacuuuAuu................   14   1   JCK-1
......................................................................................................................................................................................................................ccacagcuuucuugaacuuuUuu................   9    1   JCK-3
......................................................................................................................................................................................................................ccacagcuuucuugaacuuuAuu................   1    1   JT-2
......................................................................................................................................................................................................................ccacagcuuucuugaacuuucAu................   1    1   JCK-3
......................................................................................................................................................................................................................ccacagcuuucuugaacuuucuu................   1    0   JCK-3
......................................................................................................................................................................................................................ccacagcuuucuugaacuuuAuu................   13   1   JCK-3
......................................................................................................................................................................................................................ccacagcuuucuugaacuuuAuu................   21   1   JCK-2
......................................................................................................................................................................................................................ccacagcuuucuugaacuuuUuu................   4    1   LT-3
......................................................................................................................................................................................................................ccacagcuuucuugaacuuuAuuu...............   2    1   JCK-2
......................................................................................................................................................................................................................ccacagcuuucuugaacuuuAuuu...............   1    1   LT-2
......................................................................................................................................................................................................................ccacagcuuucuugaacuuuAuuu...............   1    1   LT-1
......................................................................................................................................................................................................................ccacagcuuucuugaacuuuUuuu...............   3    1   JCK-3
......................................................................................................................................................................................................................ccacagcuuucuugaacuuuUuuu...............   2    1   JCK-2
......................................................................................................................................................................................................................ccacagcuuucuugaacuuucAuu...............   1    1   JCK-2
......................................................................................................................................................................................................................ccacagcuuucuugaacuuuAuuu...............   1    1   LT-3
```

Star Mature

```
ucucuuucucucucucucucucucuaaaaaaacaaaaggaaagaaaaaaaaaaaaaaaaacuuacuacuacuacuaguaguauaucuucuaauuugcuggaaaggcacaccaaagagaaagaaauuaaaccaaggaaggaaggaaggaaggaaggaagugagaucugaugagagaaaaagagaauguuuuugcaaguccuggucaugcuuuuccacagcuuucuugaacuuucuuucuuucuguuuagucu
..................................................................................................................................................................................................................ccacagcuuucuugaacuuuAuuu...............   3   1   JCK-1
..................................................................................................................................................................................................................ccacagcuuucuugaacuuuUuuu...............   3   1   LT-3
..................................................................................................................................................................................................................ccacagcuuucuugaacuuucuCu...............   1   1   JCK-2
..................................................................................................................................................................................................................ccacagcuuucuugaacuuuUuuu...............   2   1   JCK-1
..................................................................................................................................................................................................................ccacagcuuucuugaacuuuUuuu...............   1   1   LT-2
..................................................................................................................................................................................................................ccacagcuuucuugaacuuuAuuu...............   1   1   JCK-3
...................................................................................................................................................................................................................cacagcuuucuugaacuu....................   1   0   LT-3
...................................................................................................................................................................................................................cacagcuuucuugaacuu....................   3   0   JCK-3
...................................................................................................................................................................................................................cCcagcuuucuugaacuu....................   1   1   LCK-3
...................................................................................................................................................................................................................cacagcuuucuugaacuu....................   3   0   LT-2
...................................................................................................................................................................................................................cacagcuuucuugaacuu....................   5   0   LCK-3
...................................................................................................................................................................................................................cacagcuuucuugaacuuA...................   2   1   JCK-3
...................................................................................................................................................................................................................cacagcuuucuugaacuuu...................   6   0   LT-3
...................................................................................................................................................................................................................cacagcuuucuugaacuuu...................   10  0   JT-3
...................................................................................................................................................................................................................cacagcuuucuugaacuuu...................   8   0   JCK-2
...................................................................................................................................................................................................................cacagcuuucuugaacuuu...................   1   0   JT-2
...................................................................................................................................................................................................................cacagcuuucuugaacuuA...................   1   1   LT-3
...................................................................................................................................................................................................................cacagcuuucuugaacuuu...................   1   0   JCK-1
...................................................................................................................................................................................................................cacagcuuucuugaacuuu...................   2   0   LT-1
...................................................................................................................................................................................................................cacagcuuucuugaacuuA...................   1   1   JCK-1
...................................................................................................................................................................................................................cacagcuuucuugaacuuu...................   4   0   LT-2
...................................................................................................................................................................................................................cacagcuuucuugaacuuu...................   2   0   JCK-3
...................................................................................................................................................................................................................cacagcuuucuugaacuAu...................   2   1   LT-2
...................................................................................................................................................................................................................cacagcuuucuugaacuuuU..................   1   1   LT-1
...................................................................................................................................................................................................................cacagcuuucuugaacuuuU..................   3   1   JCK-1
...................................................................................................................................................................................................................cacagcuuucuugaacuuuU..................   2   1   LT-3
...................................................................................................................................................................................................................cacagcuuucuugaacuuuA..................   1   1   JCK-3
...................................................................................................................................................................................................................cacagcuuucuugaacuuuU..................   5   1   JCK-2
...................................................................................................................................................................................................................cacagcuuucuugaacuuuU..................   5   1   JCK-3
...................................................................................................................................................................................................................cacagcuuucuugaacuuuA..................   1   1   LT-3
...................................................................................................................................................................................................................cacagcuuucuugaacuuuU..................   2   1   LT-2
...................................................................................................................................................................................................................cacagcuuucuugaacuuuU..................   5   1   LCK-3
...................................................................................................................................................................................................................cacagcuuucuugaacuuuA..................   4   1   LCK-3
...................................................................................................................................................................................................................cacagcuuucuugaacuuucu.................   9   0   JCK-3
...................................................................................................................................................................................................................cacagcuuucuugaacuuucu.................   2   0   JCK-2
...................................................................................................................................................................................................................cacagcuuucuugaacuuucu.................   3   0   LT-1
...................................................................................................................................................................................................................cacagcuuucuugaacuuucu.................   2   0   JCK-1
...................................................................................................................................................................................................................cacagcuuucuugaacuuuAuu................   3   1   JCK-1
...................................................................................................................................................................................................................cacagcuuucuugaacuuuAuu................   2   1   JCK-3
...................................................................................................................................................................................................................cacagcuuucuugaacuuuUuu................   2   1   LT-3
...................................................................................................................................................................................................................cacagcuuucuugaacuuuAuu................   8   1   JCK-2
...................................................................................................................................................................................................................cacagcuuucuugaacuuuUuu................   1   1   LT-1
...................................................................................................................................................................................................................cacagcuuucuugaacuuAcuuu...............   1   1   LT-1
....................................................................................................................................................................................................................acagcuuucuugaacuuu...................   1   0   LT-2
....................................................................................................................................................................................................................acagcuuucuugaacuuA...................   2   1   LT-2
....................................................................................................................................................................................................................acagcuuucuugaaUuuu...................   6   1   LCK-3
....................................................................................................................................................................................................................acagcuuucuugaacuuuU..................   3   1   LT-2
....................................................................................................................................................................................................................acagcuuucuugaacuuuA..................   1   1   LT-1
....................................................................................................................................................................................................................acagcuuucuugaacuuuU..................   1   1   LT-3
....................................................................................................................................................................................................................acagcuuucuugaacuuuU..................   4   1   LCK-3
....................................................................................................................................................................................................................acagcuuucuugaacuuuUu.................   3   1   LT-3
...................................................................................................................................................................................................................................uuucuGucuuucuguuua....   1   1   LT-2
```
